# Oscillometry bronchodilator response testing identifies distinct pathophysiological phenotypes: a *post hoc* analysis of the ATLANTIS study

**DOI:** 10.1183/23120541.00314-2026

**Published:** 2026-11-02

**Authors:** Eleanor Quek, Oliver Pitts, Franz Aaron Clemeno, Janwillem W.H. Kocks, Bianca Beghe, Monica Kraft, Ulrica Scaffidi Argentina, Alberto Papi, Klaus F. Rabe, Maarten van den Berge, Dave Singh, Christopher Brightling, Salman Siddiqui

**Affiliations:** 1 National Heart and Lung Institute, NIHR Imperial Biomedical Research Centre, Imperial College London, London, UK; 2 Data Science Institute, Imperial College London, London, UK; 3 Institute for Lung Health, National Institute for Health and Care Research Leicester Biomedical Research Centre, University of Leicester College of Life Sciences, University of Leicester, Leicester, UK; 4 General Practitioners Research Institute, Groningen, The Netherlands; 5 Department of Translational Medicine, University of Ferrara, Ferrara, Italy; 6 Department of Medicine, Icahn School of Medicine at Mount Sinai, Mount Sinai Health System, New York, NY, USA; 7 Chiesi Farmaceutici S.p.A., Parma, Italy; 8 LungenClinic Grosshansdorf, Grosshansdorf, Germany; 9 Airway Research Center North, German Center for Lung Research, Grosshansdorf, Germany; 10 Christian-Albrechts University of Kiel, Kiel, Germany; 11 Department of Pulmonary Diseases, University of Groningen, University Medical Center Groningen, Groningen, The Netherlands; 12 University of Manchester, Manchester University NHS Foundation Trust, Manchester, UK; 13 Medicines Evaluation Unit, an IQVIA business, Manchester, UK

## Abstract

**Background:**

Spirometry with bronchodilator response (BDR) testing is the first-line test for asthma in international guidelines; however, it has been shown to have suboptimal sensitivity for asthma diagnosis and detecting small airways dysfunction. Oscillometry with BDR may identify bronchodilator response in patients with no significant BDR by spirometry. The aims of the present study were to determine whether oscillometry identifies additional BDR in the Assessment of Small Airways Involvement in Asthma (ATLANTIS) study asthma cohort compared to spirometry (ClinicalTrials.gov identifier NCT02123667), and to characterise phenotypic differences between BDR groups.

**Methods:**

Baseline data from 518 adults with objectively confirmed asthma enrolled in ATLANTIS were analysed. Differences in clinical, biomarker and physiological characteristics were assessed between BDR groups. A subset with computed tomography (CT) imaging (208 out of 518) and nasal RNA-sequencing data (236 out of 518) underwent CT biomarker and differential gene expression analysis to investigate differences in airway morphology and pathways underlying a small airways bronchodilator responsive phenotype.

**Results:**

Oscillometry detected significant bronchodilator response in an additional 11% of participants not identified by spirometry, although spirometry identified more responders overall. Spirometry-positive BDR-only cases (group 1) had greater airway obstruction and exhaled nitric oxide fraction (p<0.05) compared with oscillometry-only BDR (group 2). Subgroup analysis identified differentially expressed genes related to mitochondrial stress and cell adhesion in group 3 (BDR in both) *versus* group 1 and reduced luminal area and greater gas trapping on CT imaging (p<0.05).

**Interpretation:**

Oscillometry identified additional bronchodilator-responsive participants beyond spirometry, indicating complementary physiological information. Distinct BDR patterns were associated with differing inflammatory, physiological and structural features, suggesting distinct asthma phenotypes.

## Shareable abstract

**Adding oscillometry to spirometry identifies additional bronchodilator-responsive airway disease and reveals distinct pathophysiological phenotypes in the ATLANTIS cohort** https://bit.ly/48VjajY

## Introduction

Asthma is a chronic, heterogeneous disease characterised by variable airflow obstruction, airway inflammation and airways hyperreactivity [1]. Spirometry bronchodilator response (BDR) testing is important in detecting variable airflow obstruction. However, due to the need for forced expiratory manoeuvres, it is challenging to perform [2]. Diagnostic sensitivity of spirometry BDR testing has been shown to be poor [3], and this may be due to suboptimal sensitivity in detecting small airways dysfunction.

There is increasing recognition of the importance of the small airways, which have been shown to be a major site of airway obstruction and inflammation [4, 5]. Small airways dysfunction is prevalent at all stages of severity [6], and is associated with worse asthma control and higher risk of exacerbations [7]. However, the small airways have often been described as the “silent zone”, as pathology may develop without being readily detected on routine testing [8].

Forced expiratory volume in 1 s (FEV_1_) can remain normal until as much as 75% of the small airways have become obstructed [9]. Consequently, there is increasing interest in oscillometry, a technique which measures the mechanical impedance of the respiratory system, representing the resistive and reactive forces opposing an oscillatory flow signal during tidal breathing. Resistance and reactance are derived from measured changes in pressure and flow at the mouth, with resistance reflecting airway calibre and reactance reflecting the elastic and inertial properties of the respiratory system [10]. As it requires tidal breathing, it can be performed by patients who struggle with spirometry, and with minimal operator training.

The Assessment of Small Airways Involvement in Asthma (ATLANTIS) study was the first study that aimed to evaluate which combination of physiological tests, biomarkers and imaging were best able to detect small airways dysfunction [11]. Oscillometry parameters resistance at 5 Hz (*R*_5_), resistance at 5 Hz minus 20 Hz (*R*_5–20_) and reactance at 5 Hz (*X*_5_) were found to be strong contributors to small airways dysfunction. Importantly, it showed that no single physiological test could capture small airways dysfunction, and it was hypothesised that different tests localised disease in different distributions in the bronchial tree.

Although oscillometry is increasingly used clinically, it remains an adjunct rather than a replacement for spirometry, and its superiority and necessity for determining BDR is unproven. The 2020 European Respiratory Society (ERS) technical standards proposed thresholds of −40% in *R*_5_, +50% in *X*_5_, and −80% in area of reactance (A*X*), based on 95th/5th centile responses in healthy children and adults [10], although only one adult study (Oostveen *et al*. [12], n=368) provided adequate reference data. Thamrin *et al*. [13] subsequently recommended adult-specific thresholds (*R*_5_−32% and *X*_5_+44%) drawing on the Oostveen study together with two larger healthy cohorts from Sweden (n=1495) and Australia (n=577) using different devices. However optimal oscillometry BDR thresholds remain uncertain and have not been validated across devices or populations. In this *post hoc* analysis, we aimed to evaluate whether oscillometry identifies additional BDR when applying these empirically derived thresholds and whether this characteristic identifies a clinically relevant asthma characteristic.

In light of the new unified National Institute for Health and Care Excellence (NICE)/British Thoracic Society (BTS)/Scottish Intercollegiate Guidelines Network (SIGN) asthma guidelines in the United Kingdom [14], we also aimed to explore whether oscillometry BDR provides additional physiological information beyond current first-line tests (blood eosinophil count, exhaled nitric oxide fraction (*F*_ENO_) and spirometry BDR). We further aimed to elucidate whether groups stratified by BDR represent distinct phenotypes by evaluating differences in their clinical characteristics, physiology, and inflammatory profile. A secondary objective was to assess the association between BDR pattern and prospective exacerbation risk utilising multivariable logistic regression modelling. Lastly, we aimed to explore whether different computed tomography (CT) imaging biomarkers and biological pathways underpin different BDR patterns by undertaking differential gene expression (DGE) analysis of nasal RNA-sequencing (seq) transcriptomic data.

## Methods

### Subjects

Data were analysed from the ATLANTIS study (ClinicalTrials.gov identifier NCT02123667), a prospective cohort study longitudinally assessing 773 participants with asthma and 99 healthy controls from 29 multinational centres over 1 year. Inclusion criteria included age 18–65 years and <10 pack-years smoking history. Participants with asthma had a clinical diagnosis for minimum 6 months confirmed by a chest physician as per 2012 Global Initiative for Asthma (GINA) criteria, which included positive airway hyperresponsiveness to methacholine, positive BDR (a change in FEV_1_ ≥12% and ≥200 mL), peak expiratory flow variability ≥20%, or reversibility after a cycle (*e.g.* 4 weeks) of asthma treatment. The main exclusions were a previous diagnosis of COPD or exacerbation in the previous 8 weeks. The protocol and results of the study are reported in full elsewhere [11, 15, 16].

### Study design

Participants were prospectively assessed at baseline as part of the ATLANTIS study protocol with spirometry, body plethysmography, impulse oscillometry (IOS), *F*_ENO_, multiple-breath nitrogen washout, methacholine challenge testing, quantitative CT scans (selected), nasal brushing transcriptomics (selected), blood tests, induced sputum, and questionnaires on asthma control, quality of life and health status. Data were collected for 1 year through clinic visits every 6 months and telephone follow-ups every 3 months, including documentation of exacerbations. Although healthy controls were enrolled in the original ATLANTIS study; this *post hoc* analysis focused solely on the asthma cohort as our objective was to compare oscillometry and spirometry BDR within asthma rather than *versus* healthy participants. This analysis was based on baseline data from participants that underwent full oscillometry and spirometry BDR and *F*_ENO_. The ATLANTIS study protocol was approved by the medical ethics committee at each participating centre. All participants provided written informed consent.

### Lung function tests

Spirometry was performed according to American Thoracic Society (ATS)/ERS recommendations [17] in the morning at the same time of day after bronchodilator washout. For bronchodilator response, 400 μg of salbutamol were administered by metered dose inhaler *via* spacer. Additional details are provided in the supplementary material.

IOS testing (Jaeger Masterscreen IOS) with BDR was performed according to standard procedures before spirometry [10] if the instrument was available at the site (22 out of 29 centres). IOS was performed first before other lung function tests.

### Definitions of bronchodilator response and biomarker thresholds

Prevalence of positive BDR was defined using the following criteria. Spirometry BDR: ≥10% increase in FEV_1_ or forced vital capacity (FVC) relative to predicted value in spirometry [18]. Oscillometry BDR: increase in *X*_5_ ≥44% and/or a decrease in *R*_5_ ≥32%, as per Thamrin *et al.* [13] (provenance of these thresholds are detailed in the supplementary methods). A significantly raised *F*_ENO_ was defined as ≥50 ppb, as per NICE/BTS/SIGN United Kingdom guidance [14]. As BTS/NICE/SIGN guidance does not specify a numeric eosinophil threshold, we used ≥0.3×10^9^ cells·L^−1^ in accordance with published thresholds to recognise eosinophilic asthma [19].

### CT methods

Volumetric whole-lung CT scans were undertaken in a subgroup (208 out of 518). Scans were obtained at near total lung capacity and near functional residual capacity. CT acquisition and analysis methods are described in the supplementary material.

### Nasal gene expression analysis

DGE analysis was carried out on transcriptomic data from nasal brushings. The main *a priori* analysis was conducted between group 3 (BDR in both) and group 1 (spirometry BDR only) to assess the differential expression predicating more extensive disease spanning the small and larger airways compared to larger airways alone. The Benjamini–Hochberg method was used to control the false discovery rate. Genes were considered differentially expressed with an absolute log_2_ fold change (log_2_FC) >0.25 and an adjusted p-value <0.05 and were then entered into gene enrichment pathway analysis. A correlation network analysis was carried out using Spearman's rank coefficient.

To assess the association between previously characterised asthma transcriptomic signatures and BDR patterns, we performed single-sample gene set enrichment analysis (ssGSEA). We focused on gene sets representing transcriptome-associated clusters (TACs) previously reported from the Unbiased Biomarkers for the Prediction of Respiratory Disease Outcomes (U-BIOPRED) cohort [20]: TAC1 (a T2-high eosinophilic cluster), TAC2 (a non-T2 cluster characterised by interferon-α, tumour necrosis factor (TNF)-α and inflammasome-related genes) and TAC3 (characterised by metabolic pathways, ubiquitination and mitochondrial function) (gene sets are shown in supplementary figure S2). ssGSEA was run using the GSVA package in R.

### Statistical analysis

Statistical analysis was carried out using GraphPad Prism version 10.2.2 (GraphPad Software, San Diego, CA, USA) and R (version 4.4.1). Data were assessed for normality prior to analysis. Baseline clinical characteristics were expressed as median (interquartile range) or geometric mean, or number of participants and percentage of cohort.

Participants were split into four groups: group 1 (spirometry-positive BDR only), group 2 (oscillometry-positive BDR only), group 3 (both spirometry- and oscillometry-positive BDR) and group 4 (no significant BDR in either test). The number of additional responders uniquely identified by oscillometry *versus* spirometry (in group 2) were quantified. McNemar's test was applied to the discordant classifications to assess whether the two paired tests, when applied to the same individuals, differed significantly in their classification of BDR status.

For differences between groups, ANOVA was performed with *post hoc* Tukey's test where indicated, or Chi-squared test.

Multivariable logistic regression was used to assess the association between BDR group and asthma exacerbation risk. Exacerbation was defined by need for a systemic corticosteroid course for ≥3 days, hospitalisation for asthma, and emergency department or urgent care department presentation for asthma. Covariates included sex, history of severe asthma as per GINA intensity stage 4/5, prior exacerbations, smoking status, pack-years, atopy (Phadiatop blood test positivity as a marker of IgE-mediated sensitisation) and medication adherence as measured by the eight-item Morisky Medication Adherence Scale.

## Results

### Demographics

The participant Consolidated Standards of Reporting Trials diagram is depicted in figure 1. Details of the 518 participants are provided in table 1. 55% were female and 45% were male. Most participants were Caucasian (88%) and never-smokers (76%). The median age was 45 years. Participants were distributed across all GINA treatment intensity stages.

**FIGURE 1:**
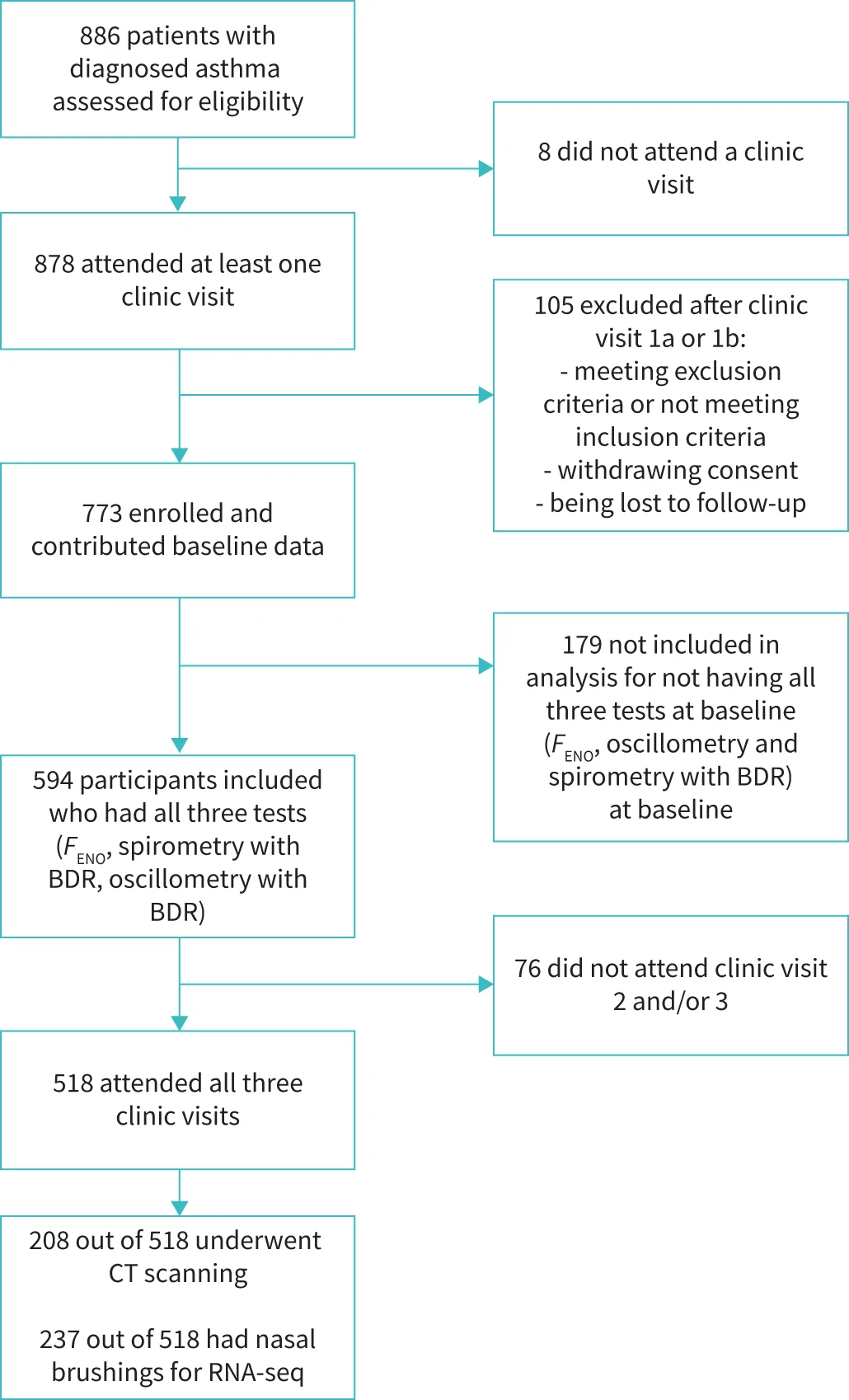
Consolidated Standards of Reporting Trials diagram. *F*_ENO_: exhaled nitric oxide fraction; BDR: bronchodilator response; CT: computed tomography; RNA-seq: RNA-sequencing.

**TABLE 1:** Baseline clinical and physiological characteristics of the Assessment of Small Airways Involvement in Asthma (ATLANTIS) asthma cohort

**Clinical characteristics**	
Age, years	45 (32–54)
Age at first diagnosis, years	24 (9–41)
Duration of disease, years	16 (6–28)
Sex	
Female	283 (55)
Male	235 (45)
BMI, kg·m^−2^	26 (23–29)
Smoking status	
Current smoker	18 (3)
Ex-smoker	108 (21)
Nonsmoker	392 (76)
GINA	
1	118 (23)
2	59 (11)
3	143 (28)
4	170 (33)
5	28 (5)
ICS use^#^	
No	118 (23)
Yes	400 (77)
Systemic corticosteroid use	
No	504 (97)
Yes	14 (3)
ACQ-6 total mean score	0.67 (0.17–1.33)
SADT score	4.98 (4.32–5.70)
Mini AQLQ total mean score	5.8 (4.9–6.4)
EQ-5D-5L	80 (75–90)
Exacerbations over previous 12 months	0 (0–0)
**Inflammatory profile**	
*F*_ENO_ (single flow at 50 mL·s^−1^), ppb	25 (16–38)
*F*_ENO_ >50 ppb	
No	434 (84)
Yes	84 (16)
Blood eosinophils ×10^9^ cells·L^−1^	0.2 (0.1–0.4)
Blood eosinophils ≥0.3×10^9^ cells·L^−1^	
No	328 (63)
Yes	185 (37)
Blood neutrophils ×10^9^ cells·L^−1^	3.6 (2.9–4.5)
Sputum eosinophils (% of nonsquamous cells)	0.4 (0.1–2.4)
Sputum eosinophils ≥3% of nonsquamous cells	
No	146 (77)
Yes	43 (23)
Sputum neutrophils % of nonsquamous cells	54 (31.5–71.3)
Sputum macrophages % of nonsquamous cells	35.2 (18.5–58.8)
Sputum lymphocytes % of nonsquamous cells	0.6 (0.3–1.5)
Phadiatop-positive (atopy)	302 (79)
**Lung physiology parameters**	
Spirometry	
FEV_1_ % predicted	85.3 (72.4–95.7)
FVC % predicted	97.5 (87.9–105.9)
FEV_1_/FVC, %	87.3 (78.1–95.0)
FEF_25–75%_ % predicted	57.9 (41.4–77.4)
FEF_50%_ % predicted	67.3 (47.8–88.3)
Body plethysmography	
TLC % predicted	103.5 (95.6–113.6)
RV/TLC % predicted	104.9 (90.1–122.7)
Oscillometry	
*X*_5_, kPa·L·s^−1^	−0.13 (−0.19– −0.09)
*R*_5_, kPa·L·s^−1^	0.43 (0.33–0.54)
A*X*, kPa·L^−1^	0.54 (0.24–1.42)
*R*_20_ kPa·L·s^−1^	0.35 (0.29–0.41)
*R*_5–20_ kPa·L·s^−1^	0.07 (0.02–0.14)
Multiple-breath nitrogen washout	
Sacin×VT L^−1^	0.11 (0.08–0.16)
Scond×VT L^−1^	0.03 (0.02–0.05)

Data are presented as median (interquartile range) or n (%). BMI: body mass index; GINA: Global Initiative for Asthma; ICS: inhaled corticosteroids; ACQ-6: six-item Asthma Control Questionnaire; SADT: Small Airways Disease Tool; Mini AQLQ: Mini Asthma Quality-of-Life Questionnaire; EQ-5D-5L: EuroQol five-dimension, five-level visual analogue scale; *F*_ENO_: exhaled nitric oxide fraction; FEV_1_: forced expiratory volume in 1 s; FVC: forced vital capacity; FEF_25–75%_: forced expiratory flow at 25–75% of FVC; FEF_50%_: forced expiratory flow at 50% of FVC; TLC: total lung capacity; RV: residual volume; *X*_5_: reactance at 5 Hz; *R*_5_: resistance at 5 Hz; A*X*: area of reactance; *R*_20_: resistance at 20 Hz; *R*_5–20_: resistance at 5 Hz – resistance at 20 Hz; Sacin×VT: ventilation homogeneity of the acinar zone of the lungs; Scond×VT: ventilation heterogeneity in the conductive zone of the lungs. ^#^: includes ICS monotherapy and ICS-containing combination therapy.

### Distribution of oscillometry BDR parameters compared to ERS recommended thresholds

The distributions of percentage changes in impedance post-bronchodilator are shown in figure 2a. Mean percentage changes in *X*_5_, *R*_5_ and A*X* were significantly lower than the thresholds recommended to identify significant BDR by Thamrin *et al*. [13].

**FIGURE 2:**
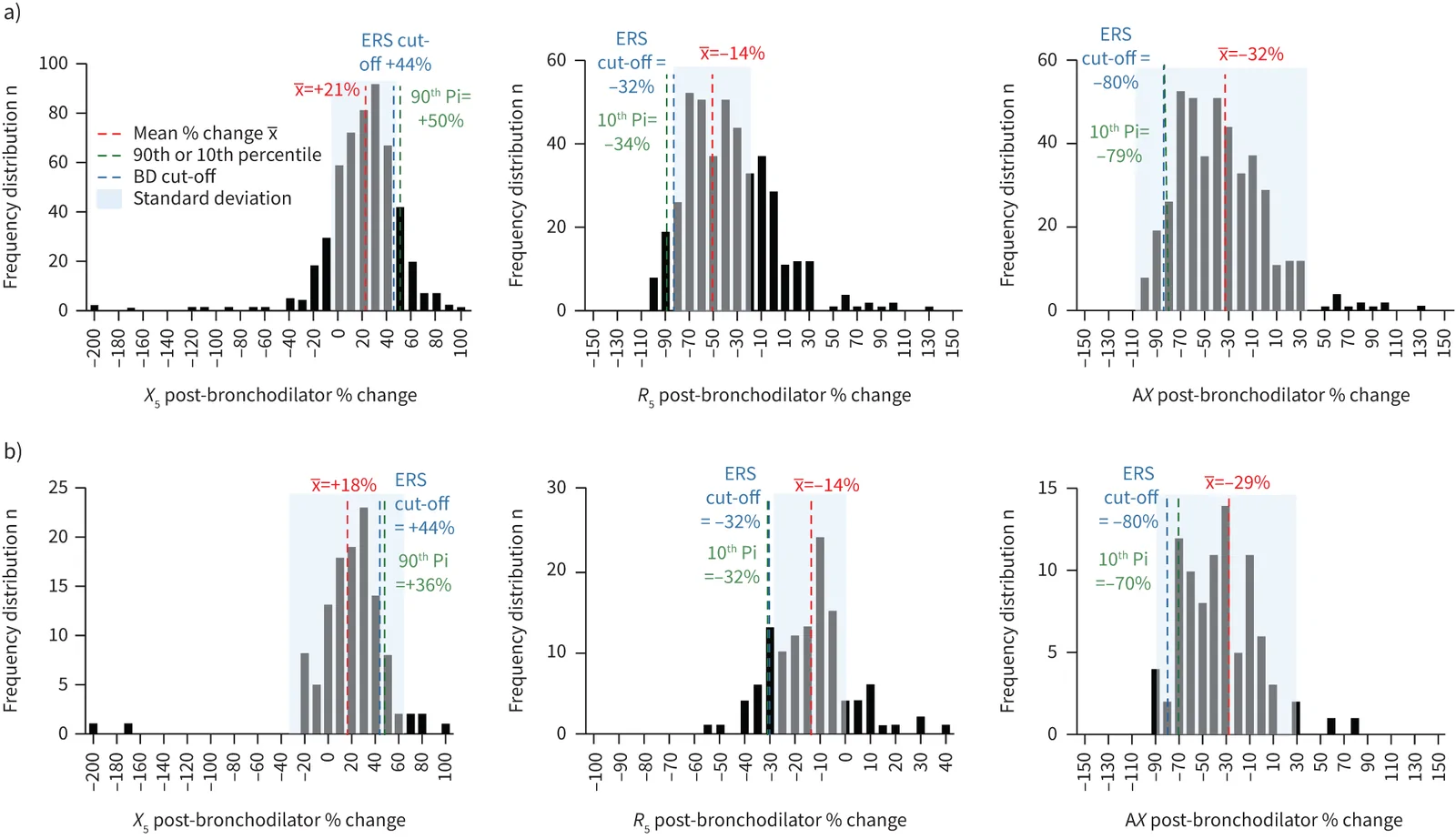
Frequency distribution of percentage change in reactance at 5 Hz (*X*_5_), resistance at 5 Hz (*R*_5_) and area of reactance (A*X*) post-bronchodilator (BD) in a) full Assessment of Small Airways Involvement in Asthma (ATLANTIS) asthma cohort and b) inhaled corticosteroid (ICS)-naïve patients only in Global Initiative for Asthma stage 1. Mean % change (x̄), 90th or 10th percentile (Pi), standard deviation are shown, as compared to the Thamrin *et al.* [13] recommended BD cut-off. Extreme values >±200% change post-BD excluded. ERS: European Respiratory Society.

To assess whether being on inhaled corticosteroids (ICS) affected oscillometry BDR, we assessed the distribution in those in GINA stage 1 who were not on ICS (figure 2b). There was no significant difference between the full asthma cohort *versus* this subgroup in percentage change in *R*_5_, *X*_5_ or A*X*.

In terms of the mode of historical diagnosis, the majority were diagnosed using methacholine challenge test (n=349, 67.4%) or with a positive spirometry BDR test (n=183, 35.3%) (supplementary figure S1a). There was a similar proportion of participants with oscillometry-detected BDR in those with positive methacholine (21%) *versus* those diagnosed with spirometry BDR (22%).

### Prevalence of bronchodilator responsiveness as defined by spirometry compared to oscillometry

Overall, 177 (34.1%) patients had positive spirometry BDR, and 110 (21.2%) patients had positive oscillometry BDR (figure 3a). Patients were split into four groups according to BDR: group 1 (n=124, 23.9%) had positive spirometry BDR only; group 2 (n=57, 11.1%) had positive BDR on oscillometry only; group 3 (n=53, 10.2%) had positive BDR in both tests; and group 4 (n=284, 54.8%) did not have significant BDR in either test (figure 3b). Oscillometry detected BDR in an additional 11.1% of participants not identified by spirometry, although overall more responders were identified by spirometry. McNemar's test indicated a significant difference in BDR classification between the two tests (p<0.001).

**FIGURE 3:**
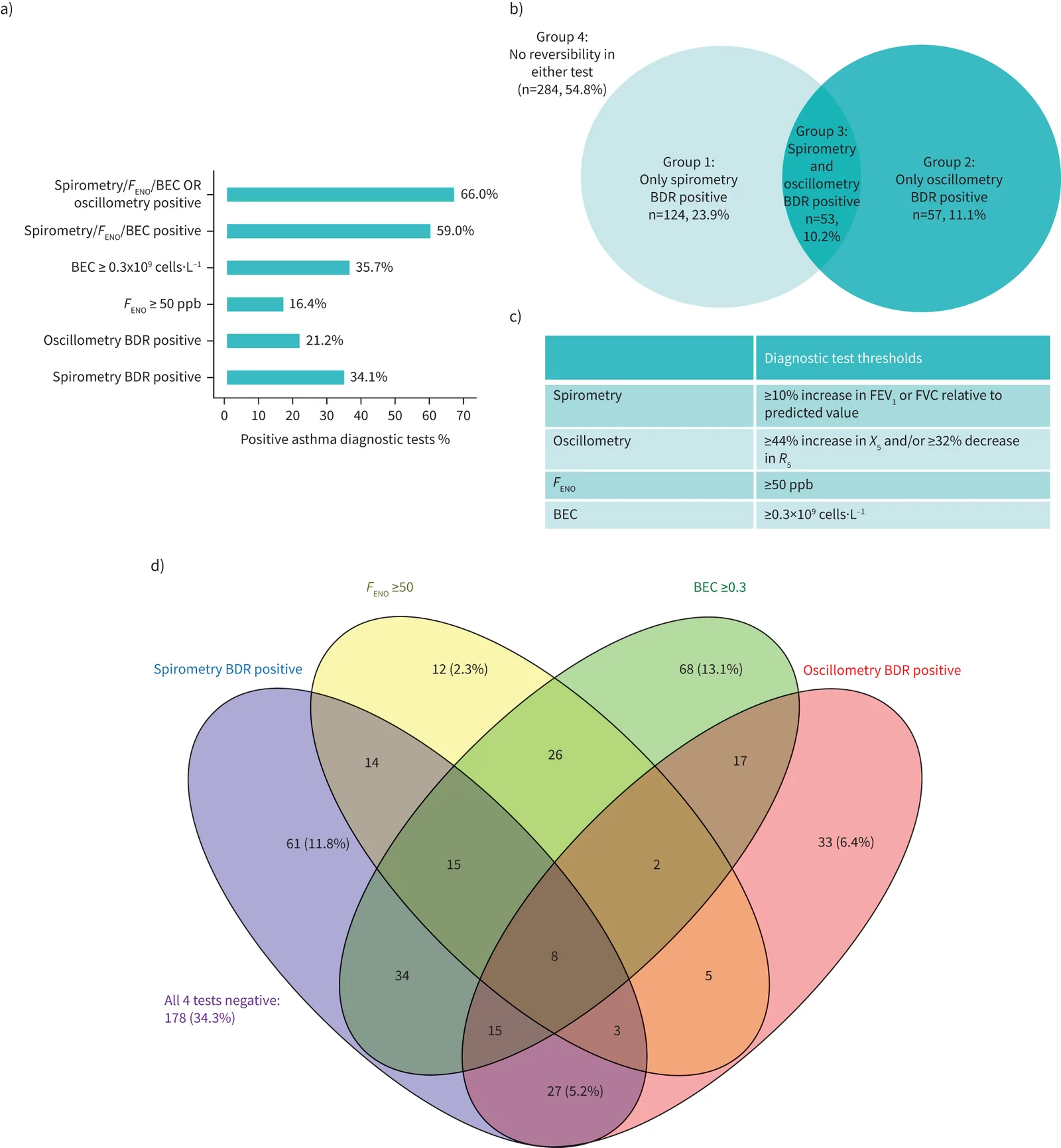
Prevalence of bronchodilator reversibility (BDR) with spirometry *versus* oscillometry. We assessed the prevalence of bronchodilator response as measured by spirometry and oscillometry within the Assessment of Small Airways Involvement in Asthma (ATLANTIS) asthma cohort. a) Proportion of positive asthma diagnostic tests including bronchodilator reversibility with spirometry and oscillometry BDR and exhaled nitric oxide fraction (*F*_ENO_) in the ATLANTIS cohort. b) Venn diagram demonstrating the proportions of participants with spirometry BDR only (group 1), oscillometry BDR only (group 2), BDR in both tests (group 3), and no reversibility in either test (group 4). c) Diagnostic test thresholds for positive BDR in spirometry and oscillometry as defined by American Thoracic Society/European Respiratory Society recommendations and positive *F*_ENO_ test as per current National Institute for Health and Care Excellence guidelines. d) Venn diagram showing concordance of spirometry BDR, *F*_ENO_, blood eosinophil count (BEC) and oscillometry BDR in the ATLANTIS asthma cohort. FEV_1_: forced expiratory volume in 1 s; FVC: forced vital capacity; *X*_5_: reactance at 5 Hz; *R*_5_: resistance at 5 Hz.

According to unified NICE/BTS/SIGN diagnostic thresholds, 307 (59.2%) out of 518 participants met one or more criterion (positive spirometry BDR, elevated *F*_ENO_ or elevated blood eosinophils) (figure 3d). Oscillometry identified significant BDR in 33 (6.4%) participants who did not meet any of these first-line test thresholds.

In a hypothetical stepwise algorithm in which oscillometry is positioned after *F*_ENO_ and blood eosinophil tests, oscillometry could identify significant BDR in 60 (11.6%) participants prior to spirometry.

### Effect of bronchodilator response status on exacerbation risk

The odds ratios and 95% confidence intervals of the effect of variables including BDR on risk of exacerbation are shown in figure 4, as computed by logistic regression modelling. Positive BDR in spirometry, oscillometry or both did not increase risk of exacerbation, alone or in combination with significantly raised blood eosinophil count or *F*_ENO_. Similarly, when modelled as individual variables, percentage changes in *X*_5_, *R*_5_ and A*X* post-bronchodilator were not associated with exacerbation risk (ORs all ≈1.00 with narrow 95% confidence intervals, p>0.10). Variables with a significant effect on exacerbation risk were history of severe asthma and previous exacerbation in the past year.

**FIGURE 4:**
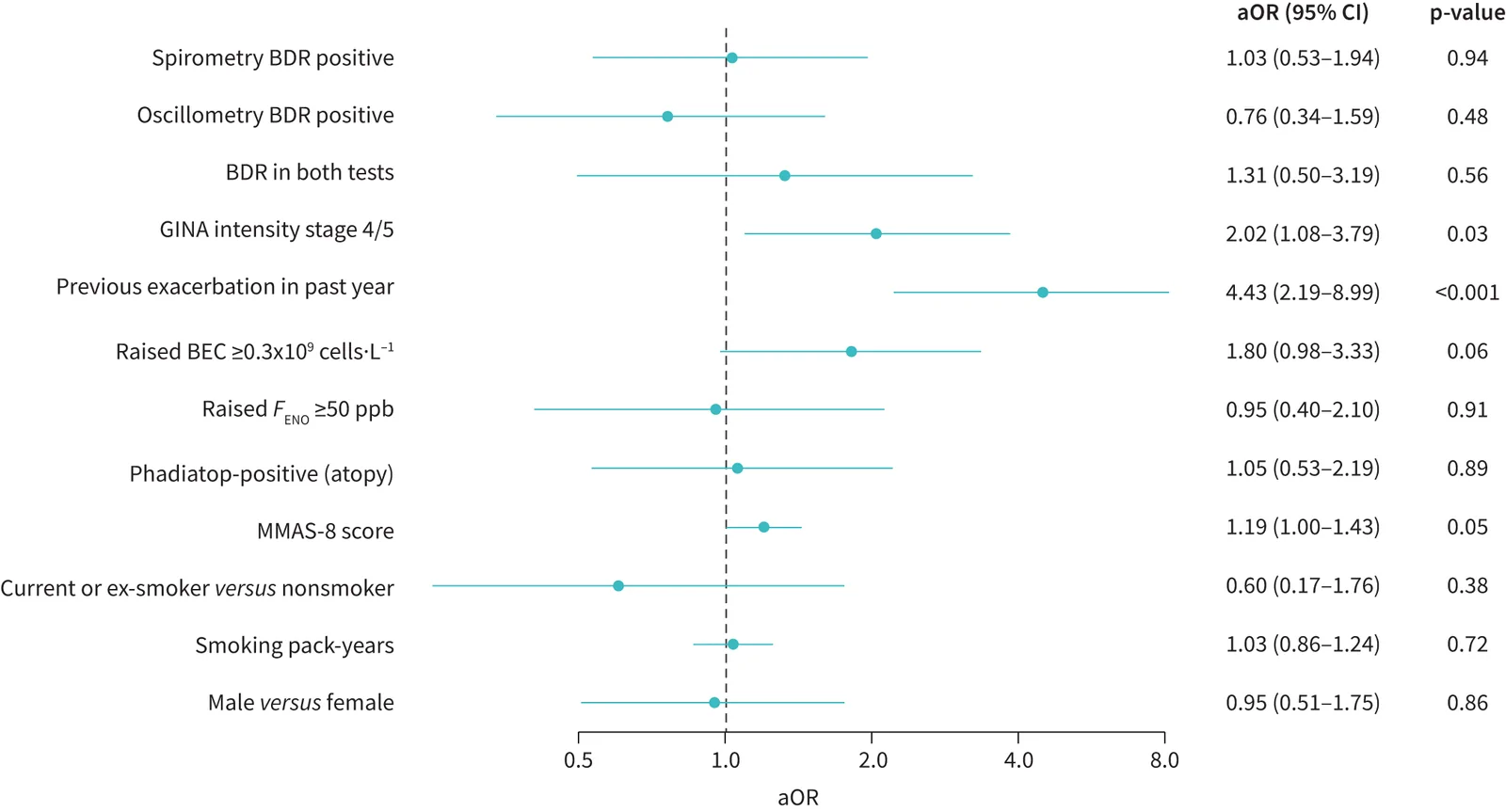
Forest plot illustrating the results of a multivariable logistic regression model assessing risk of exacerbation during 1 year of follow-up after baseline. Forest plot showing adjusted odds ratios (aORs) with 95% confidence intervals from the multivariable logistic regression model assessing predictors of exacerbation during 1-year follow-up. Covariates included bronchodilator response (BDR) (spirometry, oscillometry, or both), Global Initiative for Asthma (GINA) severity, history of previous exacerbation, raised blood eosinophil count (BEC) (≥0.3×10^9^ cells·L^−1^), raised exhaled nitric oxide fraction (*F*_ENO_) (≥50 ppb), Phadiatop positivity as a marker of IgE-mediated sensitisation (atopy), medication adherence (Morisky Medication Adherence Scale (MMAS)-8 score), smoking status, smoking pack-years, and sex.

### Differences in baseline characteristics, inflammatory profile and physiology between BDR groups

Differences in baseline characteristics are summarised in table 2. Group 3 (positive BDR in both oscillometry and spirometry) had significantly greater disease duration compared to group 4 (no significant BDR in either) (25 *versus* 14 years, p=0.04). Patients with positive BDR in both were more likely to be male (68%), while a higher proportion with BDR in neither were female (61%) (Chi-squared p<0.001). There was a significantly higher proportion of nonsmokers in group 2 (oscillometry BDR positive only) compared to other groups. There was a significantly higher proportion of patients in group 4 (BDR in neither) that were in GINA stage 4/5 compared to group 3 (positive BDR in both) (42% *versus* 30%, Chi-squared p<0.001).

**TABLE 2:** Comparison of clinical characteristics between groups with different bronchodilator responsiveness (BDR) patterns

	Group 1: spirometry BDR positive	Group 2: oscillometry BDR positive	Group 3: spirometry+oscillometry BDR positive	Group 4: no BDR	p-value
**Participants**	124	57	53	284	
**Age, years**	46 (32–54)	43 (33–53)	48 (37–54)	45 (32–54)	0.61
**Age at first diagnosis, years**	22 (7–37)	22 (7–38)	21 (7–38)	27 (10–43)	0.10
**Duration of disease (years)**	19 (8–29)	17 (6–27)	25 (10–33)^#^	14 (4–26)^¶^	**0.01**
**Sex**					
Female	68 (55)	24 (43)	17 (32)	174 (61)	**<0.001**
Male	56 (45)	32 (57)	36 (68)	110 (39)	
**BMI, kg·m^−2^**	25.4 (22.7–28.6)	26.0 (23.2–31.7)	26.4 (24.1–29.1)	25.7 (22.7–29.3)	0.49
**Smoking status**					
Current smoker	4 (3)	1 (2)	2 (4)	11 (4)	**0.02**
Ex-smoker	28 (23)	8 (14)	15 (28)	57 (20)	
Nonsmoker	92 (74)	48 (84)	36 (68)	216 (76)	
**GINA**					
1	28 (23)	11 (19)	12 (23)	67 (24)	**<0.001**
2	19 (15)	9 (16)	10 (19)	21 (7)	
3	34 (27)	17 (30)	15 (28)	77 (27)	
4	35 (28)	18 (31)	11 (21)	106 (37)	
5	8 (7)	2 (4)	5 (9)	13 (5)	
**ICS**					
No	31 (25)	12 (21)	11 (21)	64 (26)	0.58
Yes	93 (75)	45 (79)	42 (79)	220 (74)	
**Systemic corticosteroids**					
No	120 (97)	55 (96)	51 (96)	278 (98)	0.83
Yes	4 (3)	2 (4)	2 (4)	6 (2)	
**ACQ-6 total mean score**	0.66 (0.17–1.16)	1.0 (0.33–1.67)	0.67 (0.33–1.50)	0.67 (0.17–1.33)	0.06
**SADT score**	5.12 (4.25–5.79)	4.81 (4.28–5.63)	5.08 (4.47–5.66)	4.95 (4.36–5.74)	0.85
**Mini AQLQ total mean score**	5.87 (5.13–6.48)	5.4 (4.53–6.2)	6.13 (5.33–6.4)	5.64 (4.78–6.35)	0.09
**EQ-5D-5L**	85 (79–90)	80 (75–90)	80 (70–85)	83 (70–90)	0.21
**Exacerbations over previous 12 months**	0 (0–0)	0 (0–0)	0 (0–0)	0 (0–0)	0.42

Data are presented as n, median (interquartile range) or n (%), unless otherwise stated. For continuous variables, one-way ANOVA was performed with *post hoc* Tukey's honestly significant difference test where the overall ANOVA was significant. Categorical variables were compared using the chi-squared test. Bold type represents overall p<0.05. BMI: body mass index; GINA: Global Initiative for Asthma; ICS: inhaled corticosteroids; ACQ-6: six-item Asthma Control Questionnaire; SADT: Small Airways Disease Tool; Mini AQLQ: Mini Asthma Quality-of-Life Questionnaire; EQ-5D-5L: EuroQol five-dimension, five-level visual analogue scale. ^#,¶^: different superscript symbols indicate a significant pairwise difference (Tukey's *post hoc*, p<0.05); within rows subjected to *post-hoc* testing, values without a superscript were not significantly different in pairwise comparisons. No *post-hoc* testing was performed where the overall ANOVA was non-significant.

Differences in inflammatory profile were compared (table 3). Group 1 (spirometry-only positive BDR) demonstrated higher *F*_ENO_ levels when compared to group 2 (oscillometry-only positive BDR) (30 ppb *versus* 21 ppb, p=0.03), and when compared to group 4 (BDR in neither) (30 ppb *versus* 23 ppb, p=0.01). There was no significant difference in blood eosinophil count or atopic status.

**TABLE 3:** Comparison of inflammatory profile and lung physiology parameters between bronchodilator responsiveness (BDR) groups

	Group 1: spirometry BDR positive	Group 2: oscillometry BDR positive	Group 3: spirometry+oscillometry BDR positive	Group 4: no BDR in either	p-value
**Participants**	124	57	53	284	
**Inflammatory profile**					
*F*_ENO_ (single flow at 50 mL·s^−1^) ppb	30 (19–46)^#^	21 (14–34)^¶^	29 (16–40)	23 (15–35)^¶^	**0.005**
*F*_ENO_ >50 ppb					
No	95 (77)	50 (88)	42 (79)	246 (87)	0.06
Yes	29 (23)	7 (12)	10 (21)	38 (13)	
Blood eosinophils ×10^9^ cells·L^−1^	0.26 (0.16–0.37)	0.21 (0.12–0.39)	0.27 (0.14–0.38)	0.20 (0.11–0.35)	0.65
Blood eosinophils ≥0.3×10^9^ cells·L^−1^					
No	75 (60)	37 (66)	30 (58)	187 (67)	0.48
Yes	49 (40)	19 (34)	22 (42)	94 (33)	
Blood neutrophils ×10^9^ cells·L^−1^	3.50 (2.74–4.66)	3.36 (2.92–4.28)	3.59 (2.68–4.90)	3.67 (3.00–4.53)	0.90
Sputum eosinophils % of nonsquamous cells	0.6 (0.1–3.4)	0.30 (0.0–0.95)	0.40 (0.20–4.0)	0.40 (0.10–2.4)	0.67
Sputum eosinophils ≥3% of nonsquamous cells					
No	32 (73)	22 (96)	20 (69)	72 (77)	0.11
Yes	12 (27)	1 (4)	9 (31)	21 (23)	
Sputum neutrophils % of nonsquamous cells	56.3 (33.5–70.4)	45.5 (29.1–73.3)	50.8 (25.9–70.5)	54.3 (33.5–72.8)	0.95
Sputum macrophages % of nonsquamous cells	33.8 (21.6–60.0)	38.9 (21.2–59.6)	31.0 (15.2–56.8)	37. (18.5–55.2)	0.90
Sputum lymphocytes % of nonsquamous cells	0.7 (0.3–1.5)	0.4 (0.25–1.5)	0.60 (0.30–1.7)	0.60 (0.30–1.5)	0.70
Phadiatop-positive (atopy)	74 (85)	31 (75)	34 (79)	163 (78)	0.48
**Lung physiology**					
Spirometry					
FEV_1_ % predicted	79.9 (67.5–89.7)^#^	88.1 (77.3–98.3)^¶^	71.5 (61.0–81.9)^+^	91.0 (85.1–98.7)^§^	**<0.001**
FVC % predicted	99.0 (86.6–105.8)	98.6 (90.4–105.6)	93.4 (88.4–104.3)	96.8 (87.8–106.7)	0.78
FEV_1_/FVC %	0.66 (0.59–0.70)^#^	0.74 (0.67–0.79)^¶^	0.59 (0.54–0.65)^+^	0.75 (0.67–0.81)^¶,§^	**<0.001**
FEF_25–75%_ % predicted	1.58 (1.02–2.09)^#^	2.0 (1.54–2.97)^¶^	1.30 (0.87–1.70)^#^	2.23 (1.44–3.02)^¶^	**<0.001**
FEF_50%_ % predicted	1.93 (1.37–2.73)^#^	2.61 (2.14–3.64)^¶^	1.70 (1.19–2.21)^#^	2.91 (2.06–3.74)^¶^	**<0.001**
Body plethysmography					
TLC % predicted	6.33 (5.45–7.24)^#^	6.20 (5.09–7.46)^#^	7.03 (5.88–7.98)^¶^	5.77 (5.05–6.84)^#,+^	**<0.001**
RV/TLC % predicted	0.36 (0.29–0.43)	0.31 (0.27–0.39)	0.38 (0.32–0.43)^#^	0.33 (0.27–0.39)^¶^	**0.004**
Oscillometry					
*X*_5_, kPa·L·s^−1^	−0.13 (−0.2 to −0.1)^#^	−0.14 (−0.2 to −0.09)^#^	−0.2 (−0.28 to −0.13)^¶^	−0.12 (−0.16 to −0.09)^#^	**<0.001**
*R*_5_, kPa·L·s^−1^	0.44 (0.34–0.54)^#^	0.46 (0.35–0.58)^#^	0.57 (0.46–0.66)^¶^	0.39 (0.32–0.50)^+^	**<0.001**
A*X*, kPa·L^−1^	0.57 (0.22–1.24)^#^	1.09 (0.33–1.83)^#^	2.18 (0.96–3.17)^¶^	0.43 (0.21–0.89)^+^	**<0.001**
*R*_20_, kPa·L·s^−1^	0.35 (0.30–0.42)	0.36 (0.31–0.45)	0.37 (0.32–0.44)^#^	0.34 (0.28–0.40)^¶^	**0.02**
*R*_5–20_, kPa·L·s^−1^	0.07 (0.02–0.14)^#^	0.10 (0.05–0.16)^#^	0.19 (0.08–0.26)^¶^	0.05 (0.02–0.10)^+^	**<0.001**
Multiple-breath nitrogen washout					
Sacin×VT, L^−1^	0.12 (0.08–0.16)	0.10 (0.08–0.13)^#^	0.15 (0.13–0.18)^¶^	0.10 (0.07–0.16)^#^	**0.01**
Scond×VT, L^−1^	0.04 (0.02–0.06)	0.03 (0.02–0.05)	0.04 (0.03–0.06)	0.03 (0.01–0.05)	0.11

Data are presented as n, median (interquartile range) or n (%), unless otherwise stated. Comparison of inflammatory profile and lung physiology between BDR groups, as measured by exhaled nitric oxide fraction (*F*_ENO_), blood eosinophil count, blood neutrophil count, sputum cytology (expressed as percentage of nonsquamous cells), spirometry, oscillometry, body plethysmography and multiple-breath nitrogen washout. For continuous variables, one-way ANOVA was performed with *post hoc* Tukey's honestly significant difference test where the overall ANOVA was significant. Categorical variables were compared using the chi-squared test. Bold type represents overall p<0.05. FEV_1_: forced expiratory volume in 1 s; FVC: forced vital capacity; FEF_25–75%_: forced expiratory flow at 25–75% of FVC; FEF_50%_: forced expiratory flow at 50% of FVC; TLC: total lung capacity; RV: residual volume; *X*_5_: reactance at 5 Hz; *R*_5_: resistance at 5 Hz; A*X*: area of reactance; *R*_20_: resistance at 20 Hz; *R*_5–20_: resistance at 5 Hz – resistance at 20 Hz; Sacin×VT: ventilation homogeneity of the acinar zone of the lungs; Scond×VT: ventilation heterogeneity in the conductive zone of the lungs. ^#,¶,+,§^: different superscript symbols indicate a significant pairwise difference (Tukey's *post hoc*, p<0.05); within rows subjected to post-hoc testing, values without a superscript were not significantly different in pairwise comparisons. No *post-hoc* testing was performed where the overall ANOVA was non-significant.

Differences in physiology were compared between groups (table 3). Group 2 (oscillometry positive BDR only) demonstrated preserved lung function compared to group 1 (spirometry positive BDR only), with significantly higher FEV_1_ (88.1% pred *versus* 79.9% pred, p<0.001) and higher FEV_1_/FVC ratio (0.74 *versus* 0.66, p<0.001). Forced expiratory flow at 25–75% of FVC (FEF_25–75%_) also differed significantly between groups (p<0.001), with lower values in groups with spirometry-defined positive BDR (group 1 and group 3) compared to the group with oscillometry-defined positive BDR.

Group 3 (positive BDR in both) had a significantly lower FEV_1_/FVC ratio compared to all three other groups (p<0.005 for all group comparisons), and significantly lower FEV_1_ % predicted (p<0.05 for all group comparisons). Group 3 also had significantly more impaired airway impedance (*R*_5_, A*X* and *R*_5–20_) compared to all other groups, demonstrated greater ventilation heterogeneity in the acinar zone compared to group 4 (0.15 L^−1^ *versus* 0.10 L^−1^, p=0.009) and greater air trapping with significantly higher residual volume/total lung capacity ratio compared to group 4 (0.38 *versus* 0.33, p=0.01).

### CT and nasal RNA-seq subgroup analysis

A subset underwent CT scanning (208 out of 518); the baseline characteristics of this subgroup are presented in supplementary table S1. Differences in CT parameters were also computed (supplementary table S2). There was significantly higher gas trapping observed in group 3 (positive BDR in both) as measured by percentage voxels <−856 HU (VI-856) compared to group 4 (BDR in neither) (20.66 HU *versus* 11.94 HU, p=0.02). There was also a significantly lower median luminal area corrected for body surface area in group 3 (p<0.01 on *post hoc* Tukey's test compared to all other groups).

DGE analysis was carried out on RNA-seq data from nasal brushings in group 3 (BDR in both) *versus* group 1 (spirometry positive BDR control group). The baseline characteristics of this subgroup (237 out of 518) are detailed in supplementary table S3. At a threshold of log_2_FC >0.25 and p<0.05, DGE analysis revealed 26 differentially expressed genes, 17 upregulated and nine downregulated in group 3 *versus* group 1 (figure 5a). The full list of genes is summarised in supplementary table S4.

**FIGURE 5:**
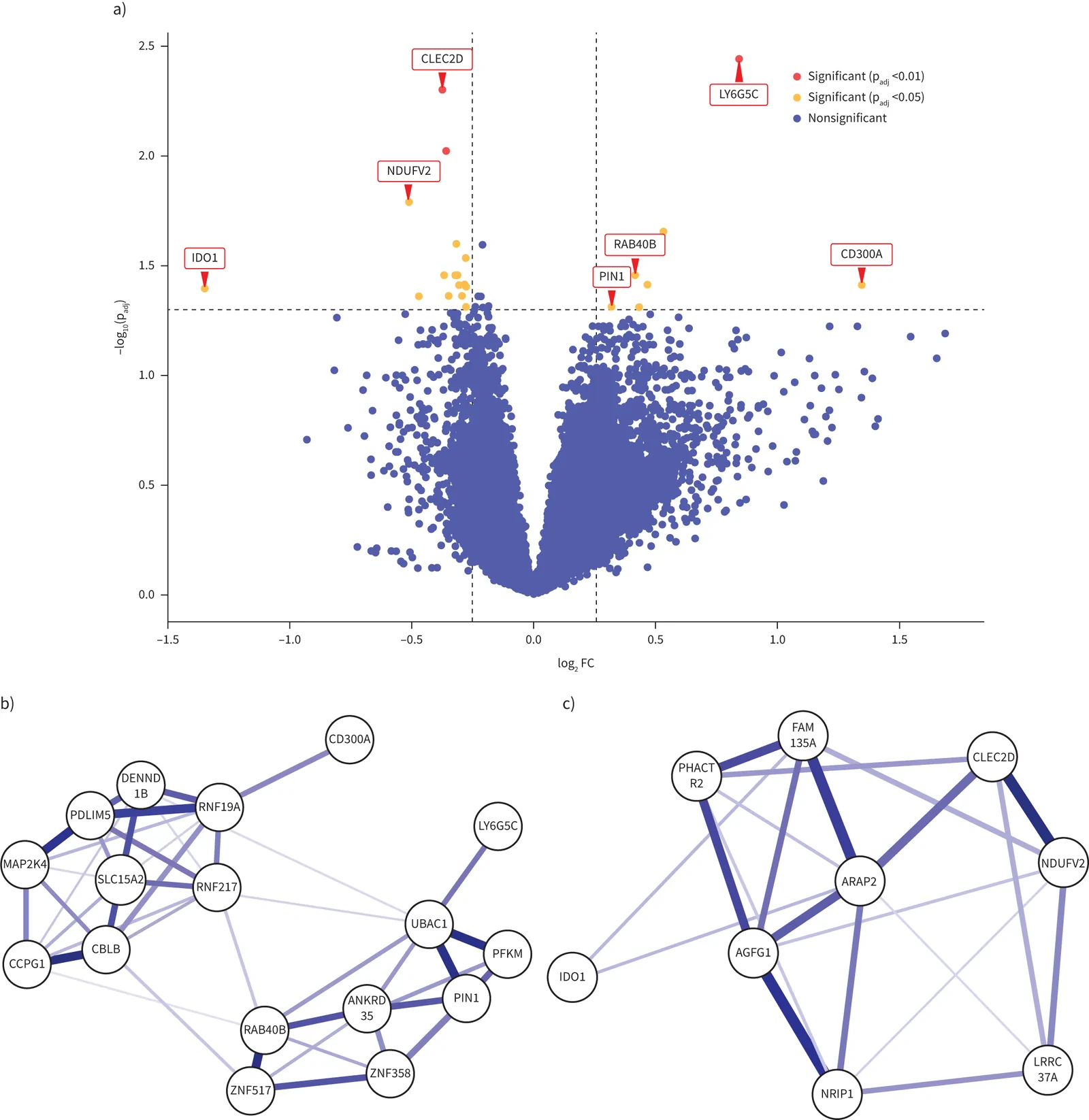
Differential gene expression in group 3 (bronchodilator response (BDR) in both spirometry and oscillometry) compared to group 1 (spirometry-only BDR). a) Volcano plot highlighting genes significantly upregulated or downregulated in group 3 *versus* group 1 (nasal brushings, 236 out of 518). Multiple testing correction was performed using the Benjamini–Hochberg method to control the false discovery rate (FDR). Upregulated genes in group 3 are on the positive axis on the right, and downregulated genes are on the negative axis on the left. Red: p<0.01 and |log_2_FC| >0.25; orange: p<0.05 and |log_2_FC| >0.25. Horizontal and vertical dashed lines indicate significance thresholds. Key genes are labelled. Relationships of genes significantly b) upregulated or c) downregulated in group 3 *versus* group 1. Network analyses were performed, where each node corresponds to a gene, while the edges (blue lines) are weighted according to the size of Spearman's rank coefficient. All edges represent positive and significant correlations (p<0.05) after FDR adjustment (p_adj_).

In pathway enrichment analysis, 17 Gene Ontology terms were significantly enriched, including pathways related to ubiquitin-mediated protein activity, mitogen-activated protein kinase activity, and cell adhesion (supplementary table S5). Downregulated pathways were related to tryptophan metabolism and mitochondrial signalling pathways (supplementary table S6). To further explore the relationships between these genes, a correlation network analysis was carried out. Figure 5b and c shows upregulated and downregulated gene networks, respectively.

There were no significant differences in the enrichment scores for any TAC gene signatures across BDR groups, suggesting that expression of these type 2 (T2)/non-T2 inflammatory transcriptomic clusters does not significantly differ by bronchodilator response phenotype in this cohort (supplementary figure S2).

## Discussion

In this *post hoc* analysis, adding oscillometry to spirometry identified additional bronchodilator-responsive patients not detected by spirometry alone. Patients stratified by pattern of BDR appear to represent distinct phenotypes with significant differences in their pathophysiological characteristics.

Oscillometry detected significant bronchodilator response in 11% of participants not identified by spirometry. However, spirometry identified more bronchodilator responders overall, suggesting that oscillometry may complement rather than replace, spirometry within a diagnostic pathway. The significant McNemar's test indicates that the two tests classify BDR differently consistent with the modalities capturing different aspects of airway mechanics. This interpretation is in in keeping with other studies that have shown lack of concordance in oscillometry- and spirometry-defined BDR [21–23], reflecting their measurement of distinct physiological domains. Where spirometry reflects larger airways obstruction and is affected by expiratory effort, respiratory muscle strength and lung volumes, oscillometry reflects resting lung mechanics and detects dysfunction in the smaller to medium-sized airways. FEF_25–75%_ is often considered a marker of small airways function, as flow at lower lung volumes is thought to be more sensitive to peripheral airways obstruction. However, it is highly dependent on lung volumes and influenced by the completeness of expiration from FVC to residual volume [24]. In this study, FEF_25–75%_ was significantly lower in groups with spirometry BDR than the group with oscillometry-only BDR, indicating that it largely aligned with conventional spirometry indices. This is consistent with previous work demonstrating that mid-expiratory flow indices did not add substantial information to the diagnosis of airflow obstruction beyond FEV_1_ and FEV_1_/FVC [25].

Oscillometry detected more subtle small airways BDR in a subgroup with normal spirometry, consistent with previous studies showing that oscillometry can detect airways disease in patients with respiratory symptoms with preserved spirometry [26–28]. The group 2 cohort appeared to have a milder disease profile with preserved FEV_1_, normal FEV_1_/FVC ratio, lower *F*_ENO_ and shorter disease duration. The higher baseline FEV_1_ in the non-BDR group (group 4) probably reflects limited capacity to demonstrate a significant BDR when baseline lung function is already normal. Oscillometry may therefore detect early small airways dysfunction in patients with normal spirometry and/or no significant BDR, although this apparent discordance may in part reflect a ceiling effect in spirometry and differences in measurement modalities rather than a true physiological separation of large and small airway function. The higher prevalence of BDR among males is consistent with recognised sex differences in asthma, in which early-life asthma is more common in boys and characterised by prominent small airways involvement that may evolve proximally [29].

Due to suboptimal sensitivity of existing first-line asthma tests, a significant proportion require bronchial challenge testing, which is resource-intensive and restricted to specialist centres [30]. Oscillometry may therefore represent a useful adjunct to detect more subtle disease missed by spirometry, potentially reducing need for bronchial challenge testing in a proportion of patients. As no independent reference standard was applied, these findings reflect additional bronchodilator responders rather than demonstrating test sensitivity; studies incorporating clinical adjudication with composite diagnostic criteria will be needed to assess diagnostic performance.

Importantly, we found oscillometry mean bronchodilator responses in this asthma cohort were significantly lower than thresholds recommended by the ERS [10] or Thamrin *et al*. [13]. There remains debate over thresholds given issues with interdevice variability, whether thresholds should be defined by healthy or asthma cohorts, or whether absolute or relative cut-offs are more suitable [13]. Further studies are needed in larger cohorts to evaluate the optimal thresholds across oscillometry parameters. A further consideration is the definition of spirometry BDR. We applied the 2022 ATS/ERS criteria (10% change in FEV_1_ or FVC relative to predicted values), which were introduced to reduce dependence on baseline lung function and to account for demographic influences including sex and height, in contrast to the 2005 criteria based on ≥12% and 200 mL change from baseline [18]. Recent evidence suggests that the use of these revised criteria may reduce prevalence of spirometry BDR, as a greater absolute change is required to meet the threshold in individuals with higher predicted lung volumes [31]. Consequently, spirometry-defined BDR may be less frequently identified using these criteria, potentially increasing the proportion of individuals classified as responsive by oscillometry alone in this cohort.

Oscillometry was able to identify patients with more extensive disease when used alongside spirometry. Group 3 (positive BDR in both) appeared to represent a more severe phenotype with worse lung function, air trapping and ventilation heterogeneity. A paediatric study also found that dual BDR was associated with worse baseline lung function than BDR in oscillometry or spirometry alone [22]. In our cohort, BDR status did not significantly increase risk of exacerbation. Multivariable models adjusting for atopy, smoke exposure and medication adherence produced similar results. Residual confounding from unmeasured environmental exposures, such as air pollution or allergen exposure, cannot be excluded. Previous studies have been conflicting, with both high and low BDR having been associated with increased risk of exacerbations [32–34]. Only 16% of participants experienced one or more exacerbation during follow-up. As such, the regression analysis may have been underpowered to detect modest prognostic effects. Future studies enriched for frequent exacerbators will be important to evaluate prognostic relevance of BDR status. The original ATLANTIS study found that pre-bronchodilator *R*_5–20_, A*X* and *X*_5_ were associated significantly with exacerbations in multivariate models [16], which may suggest that baseline abnormalities in impedance are more strongly associated with exacerbations, rather than BDR.

DGE analysis identified genes linked to pathways beyond the T2 axis. A number of genes in our study have previously been linked with asthma, including LY6G5C associated with BDR-responsive disease [35], CBLB in dust-mite induced asthma [36], CD300A as a modulator of mast cell and eosinophil function [37], RNF19A in atopy and tissue eosinophilia [38], and DENND1B which encodes a protein expressed by dendritic cells that regulates type 1 T-helper (Th1)/Th2 signalling through TNF-α-receptor-1 [39]. Network analysis identified gene modules (CBLB, RNF-19A, PDLIM5) that were enriched for pathways related to oxidative stress, most prominently ubiquitin protein activity. The process of ubiquitination regulates transcription of proteins in pro-inflammatory cascades and has been implicated in the pathogenesis of inflammatory conditions including asthma [40]. PDLIM5 (an E3 ubiquitin ligase protein) and MAP2K were also found to be upregulated and are modulators of NF-κB signalling pathways, which is known to play an important role in oxidative stress in asthma [41–43].

Those with positive spirometry BDR demonstrated higher *F*_ENO_, replicating findings in other studies [22, 44]. Although there was no significant difference in blood and sputum eosinophils, >75% of participants were receiving ICS-containing therapy, which probably suppressed T2 markers and may have attenuated inflammatory differences. The lack of association between TAC signatures and BDR further implies that small airways dysfunction may not be tightly linked to classic T2-high/Th17 molecular subtypes. Although these findings should be interpreted cautiously given the modest number of DEGs identified at the applied threshold, they are consistent with emerging concepts that distinct oxidative and epithelial stress responses may contribute to airway dysfunction beyond canonical T2-signalling.

Strengths include the large and well-characterised cohort, across a spectrum of asthma severity. As this was a group with an established asthma diagnosis that were largely already on inhaled corticosteroid-containing therapy, the prevalence of positive diagnostic test results may be underestimated as anti-inflammatory treatment may blunt bronchodilator responsiveness. As such, the diagnostic utility of oscillometry requires evaluation in treatment-naïve individuals with undiagnosed respiratory symptoms.

While bronchoscopic sampling remains impractical at scale, nasal sampling is noninvasive, and previous studies have shown that there is substantial overlap between nasal and bronchial transcriptomes, with the ability to distinguish molecular subphenotypes of asthma [45, 46]. Nonetheless, nasal tissue may not fully capture pathways specific to the distal airways and absence of TAC enrichment could reflect sampling limitations. These findings should therefore be interpreted as signal-seeking and requires validation in ICS-naïve cohorts with direct lower-airway sampling.

In conclusion, we have shown that oscillometry identified additional bronchodilator-responsive participants when used alongside spirometry. Patients stratified by pattern of BDR response appeared to represent distinct phenotypes with significantly different clinical, inflammatory and physiological traits. DGE analysis identified pathways beyond T2 biology which may elucidate new molecular targets for therapies aimed at modifying small airways dysfunction. Collectively, these findings support the concept that oscillometry provides complementary information, particularly in patients with normal spirometry. Prospective diagnostic accuracy trials should be carried out to determine the clinical utility of oscillometry as part of a multimodal assessment strategy.

## Supplementary material

**Please note:** supplementary material is not edited by the Editorial Office, and is uploaded as it has been supplied by the author. Supplementary material 00314-2026.SUPPLEMENT
