# Supplementary material for: Oscillometry bronchodilator response testing identifies distinct pathophysiological phenotypes: a post hoc analysis of the ATLANTIS study
Source: ERJ Open Res. 2026 Nov 2;12(6):00314-2026. doi: 10.1183/23120541.00314-2026 (PMC13725841; doi:10.1183/23120541.00314-2026)
Supplement: Supplementary file 1 [file 00314-2026.SUPPLEMENT.pdf]

Title: **Oscillometry bronchodilator response testing identifies distinct pathophysiological phenotypes – a post hoc analysis of the ATLANTIS study**

Authors:

Eleanor Quek, MBBS[1]

Oliver Pitts[2]

Franz Aaron Clemeno[3]

Janwillem WH Kocks, MD, PhD[4]

Bianca Beghe, MD[5]

Monica Kraft, MD[6]

Ulrica Scaffidi Argentina, MD[7]

Alberto Papi, MD[5]

Klaus F. Rabe, MD, PhD, FERS[8]

Maarten van den Berge, MD, PhD[9]

Dave Singh, MD[10]

Christopher Brightling, PhD, FMedSci[3]

Salman Siddiqui, PhD, FRCP[1]

Affiliation:

1. National Heart and Lung Institute, NIHR Imperial Biomedical Research Centre, Imperial College London, UK

2. Data Science Institute, Imperial College London, UK

3. Institute for Lung Health, National Institute for Health and Care Research Leicester Biomedical Research Centre, University of Leicester College of Life Sciences, University of Leicester, Leicester, UK

4. General Practitioners Research Institute, Groningen, Netherlands

5. Department of Translational Medicine, University of Ferrara, Ferrara, Italy

6. Department of Medicine, Icahn School of Medicine at Mount Sinai, Mount Sinai Health System, New York, NY USA

7. Chiesi Farmaceutici S.p.A., Parma, Italy

8. LungenClinic Grosshansdorf, Grosshansdorf, Germany; Airway Research Center North, German Center for Lung Research, Grosshansdorf, Germany; Christian-Albrechts University of Kiel, Kiel, Germany

9. Department of Pulmonary Diseases, University of Groningen, University Medical Center Groningen, Groningen, The Netherlands

10. NIHR Manchester Biomedical Research Centre, Manchester University NHS Foundation Trust, and University of Manchester, Manchester, UK

Correspondence:

Professor Salman Siddiqui BM, MRCP, PHD, National Heart and Lung Institute

Imperial NIHR Biomedical Research Centre (Respiratory Theme)

Imperial College, London, UK

s.siddiqui@imperial.ac.uk

Author contribution statement: EQ was responsible for idea conception, data collection and analysis and drafting figures. SS was involved in data collection, supported conception and data analysis. OP and FAC supported data analysis. CB, BB, USA, DS, JK, LMF, KFR, MvdB, MK and AP were involved in data collection. All authors were responsible for drafting all versions of the manuscript and approving the final version.

Conflicts of Interest:

EQ reports no conflicts of interest.

OP reports no conflicts of interest.

FAC reports no conflicts of interest.

JK reports grants, personal fees and non-financial support from AstraZeneca, grants, personal fees and non-financial support from Boehringer Ingelheim, grants and personal fees from Chiesi, grants, personal fees and non-financial support from GSK, non-financial support from Mundi Pharma, personal fees from Sanofi, grants and personal fees from Teva, personal fees from MSD, personal fees from COVIS Pharma, grants, personal fees from ALK-Abello, grants from Valneva; All fees go to GPRI; and Janwillem Kocks holds <5% shares of Lothar Medtec GmbH and is owner of the General Practitioners Research Institute.

BB reports payments or honoraria for lectures, presentations or speakers' bureaus from AstraZeneca, GlaxoSmithKline, Chiesi, Sanofi, Menarini Group and support for attendings meetings from Chiesi and Menarini Group

MK reports research grants paid to her institution from Sanofi, Areteia, American Lung Association and National Institutes of Health; personal fees (consulting and speaking) from Chiesi, Sanofi, Genentech, AstraZeneca, Regeneron and Kinaset, and royalties as a section editor and author for UptoDate, and is Council and Treasurer for Association of Professors of Medicine and Alliance for the Advancement of Internal Medicine (AAIM)

AP reports grants from Chiesi, AstraZeneca, GlaxoSmithKline and Sanofi; consultancy fees from Chiesi, AstraZeneca, GlaxoSmithKline, Sanofi, Avillion, Moderna, Roche, Regeneron, Zambon, Zentiva and payment or honoraria for lectures, presentations, speakers' bureaus, from Chiesi, AstraZeneca, GlaxoSmithKline, Zambon, Sanofi, Avillion, Regeneron, Moderna, Roche.

KFR has received payment or fees for lectures, presentations, speakers bureaus, manuscript writing, or educational events from AstraZeneca, Berlin-Chemie, Boehringer Ingelheim, Chiesi, GSK, Novartis, Regeneron Pharmaceuticals, Roche, and Sanofi, and has received fees for advisory board participation from AstraZeneca, Boehringer Ingelheim, Regeneron Pharmaceuticals, and Sanofi.

MvdB reports research grants paid to Institution from GlaxoSmithKline, Roche, Genentech, Chiesi, Sanofi and Astra Zeneca.

DS has received personal fees from Adovate, Almirall, Anaveon, Apogee, Arcutis Biotherapeutics, Arrowhead, AstraZeneca, Belenos Biosciences, Bial, Celldex, Chiesi, Cipla, CONNECT Biopharm, Covis. DevPro Biopharma LCC, Elpen, Empirico, EpiEndo, Generate Biomedicines, GlaxoSmithKline, Glenmark, Jasper, Kinaset Therapeutics, KOLON, Kymera, Lupin, Melodia, Menarini, MicroA, OM Pharma, OrientEuroPharma, Recipharm, Revolo, RIGImmune Inc, Roche, Roivant Sciences, Sanofi, Sitryx, Synairgen, Tetherex, UCB, Upstream, Verona Pharma, Winward, Zura Bio and Zymeworks.

CB has received grants and consultancy fees from Areteia, AstraZeneca, Chiesi, Genentech, GlaxoSmithKline, Regeneron Pharmaceuticals, Roche and Sanofi paid to his Institution.

USA is an employee of Chiesi Farmaceutici S.p.A., the funder of this work.

SS has received grants from AstraZeneca, reports advisory board and/or speaker fees from Astra Zeneca, GSK, Chiesi, Sanofi, Medscape, Areteia therapeutics, is on the scientific steering group for the ATLANTIS consortium and has participated in advisory boards for AstraZeneca, GlaxoSmithKline, Chiesi, Areteia, Sanofi and Medscape.

Word count: 4017/3000

Figures: 5

Tables: 3

References: 46

Key words: asthma; bronchodilator response; FeNO; oscillometry; spirometry; phenotype

Contents

Page 5	**Supplementary methods**
Page 7	**Figure S1**: Outcome of diagnostic tests with respect to mode of historical diagnosis and GINA severity
Page 8	**Figure S2**: Single-sample Gene Set Enrichment Analysis of Transcriptome Associated Clusters (TAC) from UBIOPRED
Page 10	**Supplementary Table S1**: Baseline characteristics of the CT subgroup
Page 12	**Supplementary Table S2**: Comparison of quantitative CT parameters between bronchodilator reversibility groups
Page 13	**Supplementary Table S3**: Baseline characteristics of nasal RNA-seq subgroup
Page 15	**Supplementary Table S4**: List of significantly differentially expressed genes in Group 3 (BDR in both) versus Group 1 (spirometry BDR only).
Page 16	**Supplementary Table S5**: List of significantly upregulated pathways in group 3 versus group 1.
Page 17	**Supplementary Table S6**: List of significantly downregulated pathways in group 3 versus group 1.
Page 18	**References**

Supplementary methods

Questionnaires

Patients completed questionnaires at baseline and 6 monthly follow up; Asthma Control Test (ACT), 6-items Asthma Control Questionnaire (ACQ-6) and mini-Asthma Quality of Life Questionnaire (miniAQLQ); EuroQol-5D-5L (EuroQol-5 dimensions-5Levels); and Small Airways Dysfunction Tool (SADT). The SADT is a 63-item questionnaire developed before and independently of the ATLANTIS study to capture signs and symptoms suggestive for SAD or less suggestive for SAD, covering domains of asthma symptoms, ear-nose-throat symptoms, localisation of somatosensory perceptions, exercise, allergens, weather conditions, fatigue, gastrointestinal complaints and skin problems[1].

Pulmonary function tests

Lung function measurements were performed according to guidelines from the ERS and ATS[2]. Predicted values were calculated according to Quanjer et al[3]. Throughout the study, the clinic visits and the lung function measurements started in the morning, approximately at the same time of the day. Participants adhered to guidelines for washout from bronchodilators: 6 hours for short acting β2-agonists (SABA); 12 hours (8 hours in patients with severe asthma defined as step 4 or 5 of treatment according to GINA 2014[4]) for short acting antimuscarinic agents (SAMA); 12 hours for long acting β2-agonists twice daily (LABA); 72 hours (24 hours in 3 patients with severe asthma defined as step 4 or 5 of treatment according to GINA 2014 (www.ginasthma.org) for long-acting β2-agonists once daily (LABA) and long-acting antimuscarinic agents (LAMA) once daily.

IOS testing (Jaeger Masterscreen IOS) with reversibility was performed according to standard procedures before spirometry [10] if the instrument was available at the site (n=22/29 centres). IOS was performed first before other lung function tests. Oscillometry BDR thresholds were defined as follows: increase in X5 ≥ 44% and/or a decrease in R5 ≥ 32% as per Thamrin et al [5]. This was derived from 95th/5th centile responses in healthy adults across three cohorts: Oostveen et al. (multi-centre study; *n* = 368; multiple devices including IOS; Jaeger, Würzburg, Germany)[6], Johansson et al. (Swedish population-based cohort; *n* = 1,495; IOS; Jaeger)[7], and Jetmalani et al. (Australian adult cohort; *n* = 577; validated in-house oscillometry device)[8]. AX was not assigned a threshold because harmonised bronchodilator centile data for AX were not reported across these reference datasets.

Spirometry was expressed as GLI z-scores. Harmonised reference equations for oscillometry and plethysmography were not available in the ATLANTIS dataset; instead, raw values were analysed and abnormality indicators (>predicted ±1.645 RSD) derived from the internal healthy control cohort were provided, consistent with the original study protocol.

FeNO was measured as per international guidelines before spirometry, at constant flow (50ml/s) in triplicate using an electrochemical/chemiluminescent sensor. The mean of 3 values within 10% of each other were used to calculate the result, and expressed as parts per billion (ppb).

Airway hyperresponsiveness was measured using a two-minute tidal breathing method or 5-breath dosimeter method according to published international guidelines[9]. Spirometry was repeated with increasing concentrations of methacholine until FEV1 decreased by at least 20% from baseline value or until the highest concentration of 16mg/ml was reached. Positive airway hyperresponsiveness thresholds were a provocative concentration when FEV1 falls 20% from baseline (PC20) <8mg/ml or

provocative dose (PD20)<0.7mg for participants not on inhaled corticosteroid (ICS), or PC20<16mg/ml or PD20<1.4mg for subjects on ICS.

Body plethysmography was performed according to standard procedures[10]. Residual Volume (RV) and RV divided by Total Lung Capacity (RV/TLC) were utilised as parameters.

Multiple-breath nitrogen washout (MBNW) was performed using EXHALYZER-D equipment according to standard procedures. Ventilation heterogeneity indices Scond and Sacin were derived from the washout slope, reflecting convection-dependent (conducting airway) and diffusion-dependent (acinar) inhomogeneity, respectively. Scond and Sacin values were analysed as provided in the ATLANTIS dataset. Units were reported as L^{-1} as per conventional ventilation heterogeneity indices. Predicted values and LLN/ULN thresholds (>predicted ±1.645 RSD) were generated from internal healthy controls as per ATLANTIS methodology.

Computed Tomography (CT)

Volumetric whole lung scans were obtained using a standardised protocol[11] for each scanner manufacturer and model to approximate to the reference scanner site (Leicester, Siemens Sensation 16 scanner [16 x 0.75 mm collimation, 1.5 mm pitch, 120 kVp, 40 mAs, 0.5 seconds rotation time and scanning field of view of 500 mm]). The scans were obtained at full inspiration (near total lung capacity) and at the end of expiration (near functional residual capacity). Prior to scanning, subjects were coached in required breath holding techniques. Participants were scanned within 1 hour of receiving 400 micrograms of salbutamol via spacer. Images were reconstructed with a slice thickness of 0.75 mm at a 0.5 mm interval using B35f kernel for the reference scanner or similar algorithm. Post processing was performed on semiautomated software, Apollo (VIDA Diagnostics, Iowa).

QCT parameters obtained included morphometry, measured in mm2, Lumen Area (LA), Total Area (TA), Wall Area (WA) ($TA - LA$) and percentage Wall Area (%WA) ($100\ x\ ((TA - LA)\ TA)$). Air-trapping measures were Mean Lung Density (MLD), the mean value of CT numbers distribution, measured upon inspiration (MLDI) , and upon expiration (MLDE), and MLD Expiratory to Inspiratory ratio (MLDE/I) measured in Hounsfield Units (HU) and Relative Voxel Change (RVC) $Exp((VI - 856) - (VI - 950)) - Insp((VI - 856) - (VI - 950))$. The Perc15 was measured in Hounsfield Units (HU), i.e. the value below which the 15% of voxels with the lowest density are distributed. Fractal dimensions of the low attenuation clusters on inspiratory scans (LAC-D -950) and on expiratory scans (LAC-D -856) were also measured. All morphometry measures were corrected for Body Surface Area mm^2/m^2 (BSA) ($\sqrt{height\ (cm)\ x\ weight\ (kg)\ 3600}$). CT scans were excluded for the following reasons: i) deviations in the CT acquisition protocol, ii) lung density was greater in the inspiratory compared to expiratory scans and iii) CT and body plethysmography lung volumes were compared and for scans where the volumes were discrepant from the mean by > 3 SD. Density measures were corrected to account for scanner manufacturer and model variability

Nasal transcriptome RNA-sequencing

Total RNA from nasal epithelium was extracted using the MiRNeasy kit from Qiagen, according to the manufacturers' instructions. Ribosomal RNA was depleted using the rRNA depletion kit (New England Biolabs, NEB#E6310). Libraries were barcoded with unique molecular identifiers (UMI) to account for duplicate counts. A pool with concentration of 1.1nM of RNA-seq libraries was used for paired-end sequencing (2×150bp) with Novaseq 6000 sequencer (Illumina, San Diego, California, USA). Raw reads quality control was performed using FastQC. Reads were mapped to the human reference genome GRCh38 with the STAR aligner [12] using gene annotation from Ensembl release 101 (ftp://ftp.ensembl.org). After alignment, UMI deduplication was done by filtering out reads with duplicate UMI barcodes and identical chromosomal locations. Concordance was checked between

reported gender and sex-chromosome-associated expression. rRNA depletion and library preparation qualities were evaluated by calculating the proportion of uniquely mapped reads.

Patient samples were grouped according to the metadata and pooled for comparisons. In RStudio, count data were prepared for differential expression analysis by generating a DGEList object. To eliminate genes with limited or inconsistent expression across samples, we applied the filterByExpr function from the edgeR package. This was guided by metadata grouping and ensured that only genes expressed in a sufficient number of samples were retained. Normalisation and differential gene expression analysis were carried out using the DESeq2 package (1.42.1), which internally adjusts for library size and sequencing depth differences across samples. Multiple testing correction was performed using the Benjamini-Hochberg method to control the false discovery rate (FDR). Genes were identified as differentially expressed based on two criteria: an absolute log2 fold change (log2FC) > 0.5 (corresponding to a fold change > 1.41) and a significance threshold of adjusted p-value (padj) < 0.05. Differentially expressed genes were then used for gene enrichment pathway analysis using GO and KEGG databases.

Figure S1. Outcome of diagnostic tests respective to mode of historical diagnosis and GINA severity. **(a)** Distribution of the mode of historical diagnosis by methacholine challenge test, spirometry BDR, peak flow variability or objective lung function response after treatment. **(b)** Outcome of baseline tests respective to historical mode of diagnosis: spirometry BDR positive, oscillometry BDR positive, FeNO≥50ppb, or any of the three being positive. **(c)** Outcome of tests respective to GINA severity groups.

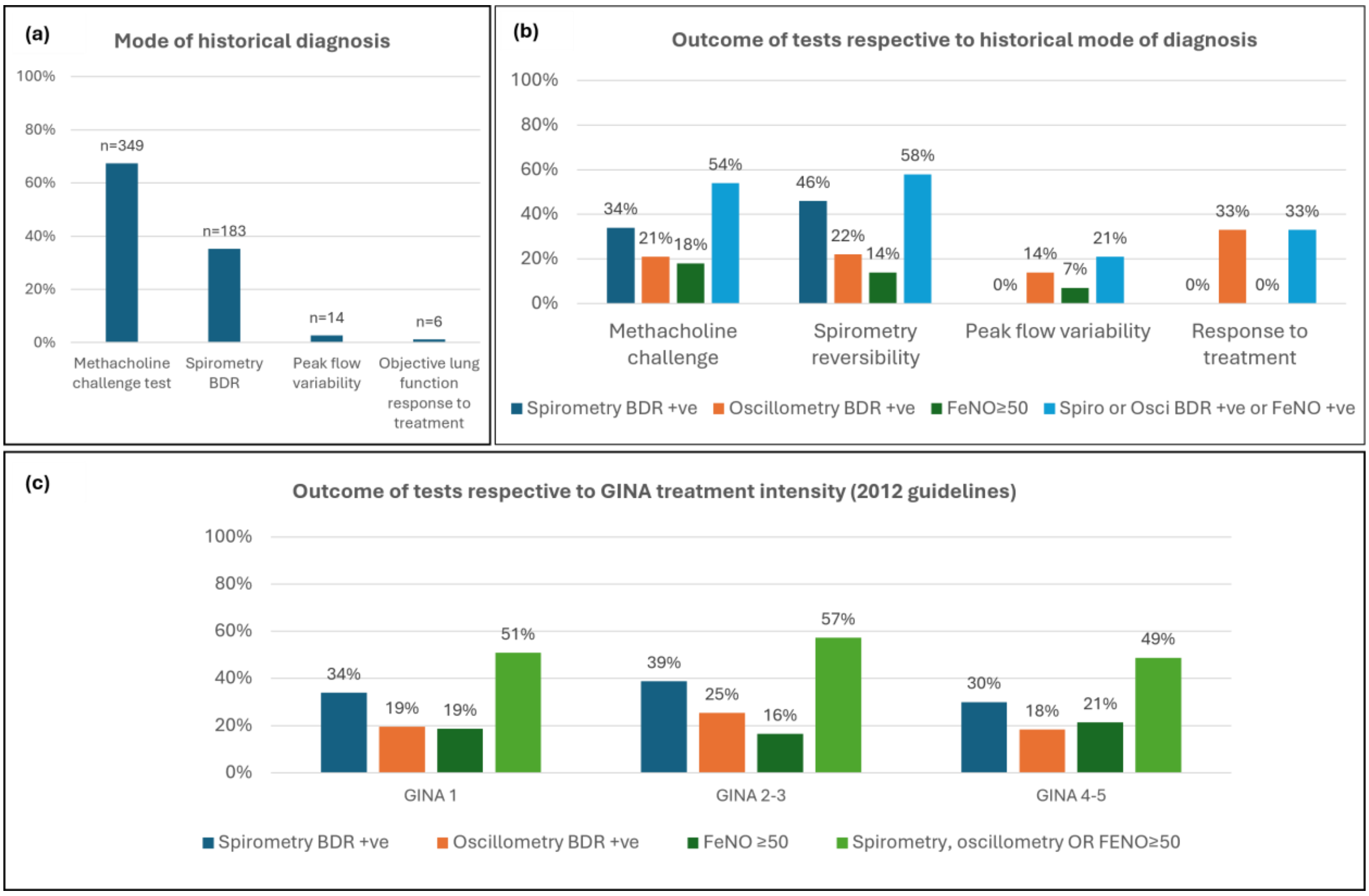

Figure S2 Single-sample Gene Set Enrichment Analysis of Transcriptome Associated Clusters (TAC) from UBIOPRED.

To assess whether *TAC1*, *TAC2*, and *TAC3* gene sets identified in the U-BIOPRED cohort are differentially expressed across bronchodilator reversibility (BDR) subgroups, gene expression data were first normalized, and single-sample Gene Set Enrichment Analysis (ssGSEA) were performed using the predefined gene sets. Samples were assigned to one of four BDR groups based on clinical metadata. Enrichment scores were visualized using box, and statistical differences between groups were tested using the Kruskal-Wallis test. (a) Enrichment scores are shown using boxplots, with comparison made using Kruskal-Wallis test. (b) List of gene sets in each transcriptome associated cluster.

(a)

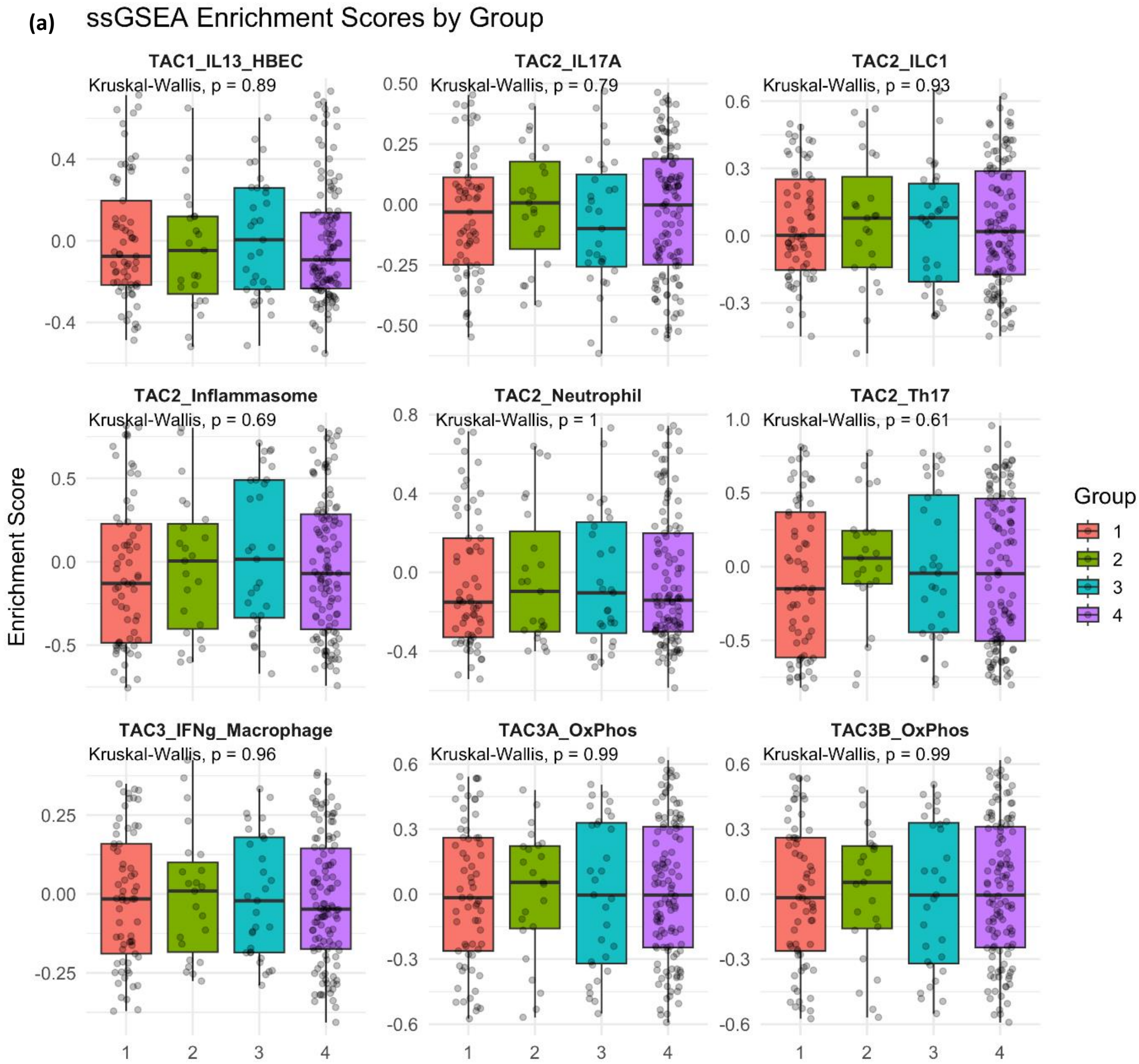

(b)

Cluster	Gene set	Genes
**TAC1**	IL-13 stimulated HBEC gene set	CST1, CCL26, PRB2, PRB1, PRB3, POSTN, PRB4, ITLN1, ALOX15, SH2D1B, CA2, NOS2, FCGBP, FOXA3, SPDEF, CAPN14, DUOXA2, CLDN5, PADI3, TSPAN8, ALPL, KCNJ16, FETUB, B3GNT6, CDH26, LRRC31, MUC13, VSIG2, CSTA, FAM3B, SLC9B2, NTRK1, KLF4, HPDL, SOCS1, TRNP1, HS3ST1, VWF, DUOX2, CISH, ATP13A5, ZNF808, RNASE4, CCBL1, SDCBP2, TMPRSS2, HYAL1, CCDC109B, FAM83D, TRAK1, TPK1, SLC7A1, CYP2C18, CDC42EP5, KCNS3, ADRA2A, MRAP2, SLC2A10, PPARG, FAM26E, ADCY4, WNT3, SLCO4A1, ALDH1A2, C10orf99, WDFY2
**TAC2**	ILC-1 cell gene set	SIT1,CD3D,CD3G,CD4,CD6,TRAV131,CD5,CD27,C14orf64,COTL1,CD8A,IKZF3,LITAF,CCR7,TRAV8-2, TRAV4, SYNE2..1, MAL, GZMM, GZMK, TC2N, GZMA, SH2D1A, IFNG-AS1, PASK, TRBV5-1, ADTRP, TRAV92, CACNA1I, CCL5, ACTN1, CXCR3, BCL11B, PYHIN1, CH25H, LBH, FBLN7, LINC00402, TRAV2, TRBV2, IGFBP3, ANK3, IL6R, LDLRAP1, ACSL6, TRAV41, MIAT, TRBV20-1, LAG3, IFNG, TRAV26-2, GABBR1, TSHZ2, SLC25A4, AP000569.8..1, RASGRF2, TRAV8-4, RP11 664D1.1, TNFRSF10D, PLEKHB1, TRAV12-2
	Neutrophil activation gene set	ABTB1, AMPD2, C5orf6, CCR3, CDA, CKLFSF2, CLC, CREB5, CTBS, DcR1, EST, FCGR2B, FCGR3B, FLJ10298, FPRL1, FRAT2, GPR27, GPR43, HSPA6, IL8RA, IL8RB, KIAA0779, KIAA1126, KRT23, LENG4, LENG5, MAD, MGC10500, MGC14126, MGC16353, MPPE1, MSCP, NCF4, NRBF2, PHC2, PROK2, RALB, RNF141, SEC14L1, SEPX1, STX3A, TM4-B, VMP1, VNN2, XPO6
	Inflammasome gene set	IL1B, NLRP3, CASP1, CASP4, CASP5
	Th17 cell gene set	CXCL1, CXCL2, CXCL3, CXCL8, CSF3
	IL17A gene set	NFKBIZ, ZC3H12A, RELB, BIRC3, CXCL1, ZC3H12C, TNFAIP3, MAP3K8, CXCL2, CASZ1, IER3, LENG1, SOD2, RRAD, NFKB2, ID2, TATDN1, CBR3, CCL3, ICAM1, PIK3R6, GADD45B, ADAM21, ZBTB25
**TAC3A**	IFN-Y Stimulated Macrophages gene set	SELM, EHD1, IL10RA, NAMPT, MCTP1, C15orf48, SLC7A11, SIGLEC10, CDK5RAP2, EREG, TERF2IP, IL1A, ISG20, MYH11, MCOLN2, CLCF1, DDIT4, ANKRD12, AARS, SQSTM1, FLNB, NUB1, DCUN1D3, CXCL8, RILPL2, DRAM1, ATOX1, PTGS2, MAP3K4, UPB1, ADARB1, CREG1, PTPN1, RNF19B, IER3, NKG7, FAM60A, IL2RA, SLC7A1, MT1H, SAMSN1, ASCC1, FYN, MT2A, ETS1, PPP3CC, MT1A, LAT, RHBDF2, G0S2, CEP135, PANX2, C5orf15, HMGN2P46, ORM1, TRAF1, C17orf58, MB21D2, LPAR1, TRAF3IP2, TNFAIP3, SEMA4A, ARHGEF2, LMO4, TNIP1, TBC1D9, ACSL5, CYP2S1, BTG3, TNIP3, EXOG, NLRP3, SEMA6B, PSTPIP2, PTGES, MARCKSL1, CD86, NFKB1, LSS, AQP9, TANK, CCR7, MT1G, LAMB3, SLC25A37, RUNX2, ADAMDEC1, NFKBIE, SYT11, PVRL2, MLLT6, MT1F, ETS2, SLC7A5, CA12, MRPS6, GK, SLC3A2, BHLHE41, WARS, TNFAIP2, CXCL16, PILRA, CCDC115, TMEM205, HIST2H2BE, SIK3, ITGB8, PPP1R18, PTPRE, NBN, HIVEP1, GRAMD1A, KYNU, SLC1A3, NFE2L1, PCNX, CD14, CYP27B1, APBB3, FPR2, CEP350, CTTN, IRAK2, DDX59, CCM2L, PIM2, TSPAN33, ACO1, TAP1, RNF144B, CXCL5, IKBKE, EMR3, APOL3, H1F0, NADK, ZMIZ2, NFS1, HLAF, BATF3, HIF1A, MTF1, YARS, STOM, RASGRP1, HIST2H2AC, GJB2, SLAMF1, CLEC4E, CCL5, KIAA0226L, GNA15, CFB, AMPD3, PNKD, MT1X, SMOX, IL1B, BIRC3, MAP1LC3A, ZMYND15, SLC2A6, SPECC1L, OSM, PRKCH, C1S, LAD1, PDE4B, STAT4, GPR84, TNFAIP6, C1orf122, PLAGL2, SLC43A2, HELZ2, TSC22D1, SLC5A3, CXCL1, HEY1, ABHD17C, CRISPLD2, DNAJB6, PGD, NDP, MMP9, ASNS, IRF7, ACKR3, RCN1, SLC9A1, RFTN1, EBI3, TGFA, PPA1, ADORA2A, CLEC4D, MUCL1, GPR132, RELB, FAM20A, GCH1, CTSL, NINJ1, ST3GAL1, SMAP2, MYO1B, TYMP, TNFRSF4, NCF1C, GBP5, HBEGF, CYB561A3, MT1IP, ZSWIM4, IL36G, LAMP3, MT1E, ZHX2, PFKFB3, N4BP1, MXD1, GNA12, CD38, PARP10, DTX2, LILRB2, TMEM132A, EMR2, SPHK1, SIRPA, PLAUR, VEGFA, PTPRF, C1QTNF1, STEAP3, MPZL1, CD82, CLEC4A, DUSP10, GLIS3, SOD2, ISCU
	Oxidative phosphorylation gene set	ND1, ND2, ND3, ND4, ND4L, ND5, ND6, NDUFS1, NDUFS2, NDUFS3, NDUFS4, NDUFS5, NDUFS6, NDUFS7, NDUFS8, NDUFV1, NDUFV2, NDUFV3, NDUFA1, NDUFA2, NDUFA3, NDUFA4, NDUFA4L2, NDUFA5, NDUFA6, NDUFA7, NDUFA8, NDUFA9, NDUFA10, NDUFAB1, NDUFA11, NDUFA12, NDUFA13, NDUFB1, NDUFB2, NDUFB3, NDUFB4, NDUFB5, NDUFB6, NDUFB7, NDUFB8, NDUFB9, NDUFB10, NDUFB11, NDUFC1, NDUFC2, NDUFC2, KCTD14, SDHA, SDHB, SDHC, SDHD, UQCRFS1, CYTB, CYC1, UQCRC1, UQCRC2, UQCRH, UQCRHL, UQCRB, UQCRQ, UQCR10, UQCR11, COX10, COX3, COX1, COX2, COX4I2, COX4I1, COX5A, COX5B, COX6A1, COX6A2, COX6B1, COX6B2, COX6C, COX7A1, COX7A2, COX7A2L, COX7B, COX7B2, COX7C, COX8C, COX8A, COX11, COX15, COX17, ATP5A1, ATP5B, ATP5C1, ATP5D, ATP5E, ATP5O, ATP6, ATP5F1, ATP5G1, ATP5G2, ATP5G3, ATP5H, ATP5I, ATP5J2, ATP5L, ATP5J, ATP8, ATP6V1A, ATP6V1B1, ATP6V1B2, ATP6V1C2, ATP6V1C1, ATP6V1D, ATP6V1E2, ATP6V1E1, ATP6V1F, ATP6V1G1, ATP6V1G3, ATP6V1G2, ATP6V1H, TCIRG1, ATP6V0A2, ATP6V0A4, ATP6V0A1, ATP6V0C, ATP6V0B, ATP6V0D1, ATP6V0D2, ATP6V0E1, ATP6V0E2, ATP6AP1, ATP4A, ATP4B, ATP12A, PPA2, PPA1, LHPP
**TAC3B**	Oxidative phosphorylation gene set	ND1, ND2, ND3, ND4, ND4L, ND5, ND6, NDUFS1, NDUFS2, NDUFS3, NDUFS4, NDUFS5, NDUFS6, NDUFS7, NDUFS8, NDUFV1, NDUFV2, NDUFV3, NDUFA1, NDUFA2, NDUFA3, NDUFA4, NDUFA4L2, NDUFA5, NDUFA6, NDUFA7, NDUFA8, NDUFA9, NDUFA10, NDUFAB1, NDUFA11, NDUFA12, NDUFA13, NDUFB1, NDUFB2, NDUFB3, NDUFB4, NDUFB5, NDUFB6, NDUFB7, NDUFB8, NDUFB9, NDUFB10, NDUFB11, NDUFC1, NDUFC2, NDUFC2, KCTD14, SDHA, SDHB, SDHC, SDHD, UQCRFS1, CYTB, CYC1, UQCRC1, UQCRC2, UQCRH, UQCRHL, UQCRB, UQCRQ, UQCR10, UQCR11, COX10, COX3, COX1, COX2, COX4I2, COX4I1, COX5A, COX5B, COX6A1, COX6A2, COX6B1, COX6B2, COX6C, COX7A1, COX7A2, COX7A2L, COX7B, COX7B2, COX7C, COX8C, COX8A, COX11, COX15, COX17, ATP5A1, ATP5B, ATP5C1, ATP5D, ATP5E, ATP5O, ATP6, ATP5F1, ATP5G1, ATP5G2, ATP5G3, ATP5H, ATP5I, ATP5J2, ATP5L, ATP5J, ATP8, ATP6V1A, ATP6V1B1, ATP6V1B2, ATP6V1C2, ATP6V1C1, ATP6V1D, ATP6V1E2, ATP6V1E1, ATP6V1F, ATP6V1G1, ATP6V1G3, ATP6V1G2, ATP6V1H, TCIRG1, ATP6V0A2, ATP6V0A4,

		ATP6V0A1, ATP6V0C, ATP6V0B, ATP6V0D1, ATP6V0D2, ATP6V0E1, ATP6V0E2, ATP6AP1, ATP4A, ATP4B, ATP12A, PPA2, PPA1, LHPP

Table S1: Baseline clinical, physiological, and CT characteristics of CT subgroup

Data are expressed as median (IQR), n (%).

Clinical characteristics of CT subgroup	N=208/518
Age (years)	46 (30 to 56)
Age at first diagnosis (years)	19 (6-40)
Duration of disease (years)	20 (8-30)
Sex	
Female	120 (58%)
Male	88 (42%)
BMI (kg/m^2)	26 (23 to 30)
Smoking status	
Current smoker	8 (4%)
Ex-smoker	47 (22%)
Non-Smoker	153 (74%)
GINA	
1	35 (17%)
2	35 (17%)
3	56 (27%)
4	72 (35%)
5	10 (3%)
Inhaled corticosteroids	
No	39 (19%)
Yes	169 (81%)
Systemic corticosteroids	
No	202 (97%)
Yes	6 (3%)
ACQ-6 total mean score	0.67 (0.17-1.33)
SADT score	5.12 (4.40 - 5.75)
MINI AQLQ total mean score	5.9 (5.2-6.5)
EuroQoL 5D-5L	80 (75-90)
Exacerbations over previous 12 months median(IQR)	0 (0-0)
**Inflammatory profile**	
FeNO (single flow at 50ml/s) (ppb)	27 (17-41)
FeNO > 50ppb	
No	170 (82%)
Yes	38 (18%)
Blood Eosinophils (10^9/L)	0.2 (0.1-0.4)
Blood Eosinophils (10^9/L)≥0.3	
No	125 (60%)
Yes	83 (40%)
Blood neutrophils (10^9/L)	3.6 (2.8-4.4)
Sputum Eosinophils (% of non-squamous cells)	0.6 (0.1 - 3.3)
Sputum Eosinophils (% of non-squamous cells)≥3	
No	67 (73%)
Yes	25 (27%)

**Sputum Neutrophils (% of non-squamous cells)**	48.7 (24.2 - 69.2)
**Sputum Macrophages (% of non-squamous cells)**	41.2 (19.6 - 61.5)
**Sputum lymphocytes (% of non-squamous cells)**	0.6 (0.2 - 1.1)
**Lung physiology parameters**	
**Spirometry**	
**FEV1 % of predicted normal value**	86.0 (71.7 to 96.7)
**FVC (% predicted of normal value)**	98.5 (88.7 - 110.1)
**FEV1/FVC (%)**	0.70 (0.63 to 0.76)
**FEF2575 (% of predicted normal value)**	58.3 (41.3 to 75.6)
**FEF50 (% of predicted normal value)**	66.8 (47.5 to 86.9)
**Body plethysmography**	
**TLC (L) (% of predicted normal value)**	103.1 (95.3 to 114.4)
**RV/TLC (% of predicted normal value)**	99.7 (85.9 to 119.0)
**Oscillometry**	
**X5 (kPa/L/s)**	-0.15 (-0.21 to -0.1)
**R5 (kPa/L/s)**	0.45 (0.34 - 0.56)
**AX (kPa/L)**	0.67 (0.3 to 1.73)
**R20 (kPa/L/s)**	0.35 (0.30 - 0.42)
**R5-20 (kPa/L/s)**	0.07 (0.02 to 0.15)
**Multiple breath nitrogen washout**	
**Sacin*VT (L^{-1})**	0.10 (0.07 to 0.16)
**Scond*VT (L^{-1})**	0.04 (0.02 to 0.05)
**Quantitative CT**	
**Expiratory MLD (Hounsfield Units (HU))**	-686.4 (-738.6 to -640.3)
**Inspiratory MLD (HU)**	-843.1 (-859.1 to -815.5)
**Expiratory to inspiratory MLD ratio**	0.82 (0.77 - 0.88)
**Expiratory Voxel index (VI) at -856 HU**	8.49 (2.65 - 19.67)
**Inspiratory VI at -950 HU**	3.99 (1.77 - 8.42)
**Median inspiratory % WA/TA**	62.4 (60.3 - 64.9)
**Median inspiratory LA/BSA (mm^2)**	10.6 (8.7 - 12.3)
**Median inspiratory TA/BSA (mm^2)**	28.6 (25.5 - 33.2)
**Median inspiratory WA/BSA (mm^2)**	17.6 (15.6 - 20.3)
**Inspiratory Pi10 (mm)**	7.1 (6.5 - 7.8)
**Inspiratory Po20 (mm)**	7.4 (6.7 to 8.5)
**15th percentile inspiratory (HU)**	-922.48 (-936.8 to -909.7)
**Expiratory lung volume (mL)**	2625.7 (2141.9 - 3156.4)
**Inspiratory lung volume (mL)**	5263.6 (4509.9 - 6356.3)
**Expiratory to inspiratory lung volume ratio**	0.50 (0.43 - 0.58)

Table S2. Comparison of quantitative CT parameters between bronchodilator reversibility groups

Only a selected cohort of patients underwent CT scanning at specific centres (N=208/518). For differences between groups, one-way analysis of variance (ANOVA) was performed with post-hoc Tukey's HSD test where indicated. Overall ANOVA p-values<0.05 are shown in bold. Values sharing a common superscript letter are not significantly different (Tukey's post hoc, p<0.05). Where no superscript appears, no post hoc differences were detected. Expiratory MLD=expiratory mean lung density. Inspiratory MLD=inspiratory mean lung density. Expiratory VI-856 - expiratory voxel index (at –856 Hounsfield units or less). Inspiratory VI-950 =inspiratory voxel index (at –950 Hounsfield units or less). WA=wall area. TA= total airway area. BSA=body surface area. Percentile 15 inspiratory=the Hounsfield Unit at which 15% of the voxels fall below. Inspiratory Pi10=10-mm internal luminal perimeter. Inspiratory Po20=20-mm external luminal perimeter.

	Group 1: Spirometry BDR positive (N = 46/124)	Group 2: Oscillometry BDR positive (N = 25/57)	Group 3: Spirometry + Oscillometry BDR positive (N=23/53)	Group 4: No reversibility (N = 114/284)	p-value
	Mean ± SD or Median (IQR)	Mean ± SD or Median (IQR)	Mean ± SD or Median (IQR)	Mean ± SD or Median (IQR)	
Expiratory MLD (Hounsfield Units (HU))	-692.2 (-735.5 to -639.5)	-667.2 (-721.7 to -632.2)	-735.3 (-773.9 to -676.1)	-684.4 (-733.2 to -635.4)	0.74
Inspiratory MLD (HU)	-838.26 ± 33.17	-833.7 ± 36.90	-840.17 ± 26.22	-829.49 ± 44.88	0.40
Expiratory to inspiratory MLD ratio	0.81 ± 0.09	0.81 ± 0.07	0.86 ± 0.07	0.82 ± 0.08	0.52
Expiratory Voxel index (VI) at -856 HU	12.40 ± 12.47	13.79 ± 17.86	20.66 ± 15.52[a]	11.94 ± 13.37[b]	**0.04**
Inspiratory VI at -950 HU	6.61 ± 6.27	6.40 ± 6.39	6.03 ± 3.72	5.25 ± 5.10	0.45
Median inspiratory % WA/TA	62.36 (59.91 - 63.84)	62.95 (61.17 - 65.84)	62.19 (60.43 - 65.38)	62.46 -(60.36 - 64.77)	0.79
Median inspiratory LA/BSA (mm^2)	10.34 (7.52 - 12.45)[a]	10.77 (8.67 - 11.84)[a]	10.18 (8.86 - 10.97)[b]	10.61 (9.00 - 12.37)[a]	**<0.001**
Median inspiratory TA/BSA (mm^2)	29.19 (23.69 - 33.25)	27.68 (25.82 - 31.34)	28.20 (26.67 - 30.61)	28.60 (25.46 - 34.07)	0.84
Median inspiratory WA/BSA (mm^2)	17.76 (14.79 - 20.54)	17.26 (16.65 - 19.17)	16.63 (15.51 - 19.89)	17.77 (15.86 - 20.63)	0.78
Inspiratory Pi10 (mm)	7.34 (6.71 - 7.92)	7.45 (6.86 - 8.03)	6.87 (6.27 - 7.93)	7.09 (6.27 - 7.77)	0.18
Inspiratory Po20 (mm)	7.13 (6.64 - 8.65)	7.38 (6.89 - 8.75)	6.98 (6.39 - 8.30)	7.71 (6.75 - 8.54)	0.45
15th percentile inspiratory (HU)	-925.05 ± 24.39	-920.66 ± 27.33	-925.85 ± 15.63	-916.72 ± 26.65	0.80
Expiratory lung volume (mL)	2699.78 ± 922.11	2769.54 ± 1356.56	3236.26 ± 1132.07	2651.06 ± 769.42	0.10
Inspiratory lung volume (mL)	5598.05 ± 1237.82	5704.61 ± 1772.91	6015.42 ± 1603.26	5277.28 ± 1361.68	0.27
Expiratory to inspiratory lung	0.48 ± 0.13	0.49 ± 0.13	0.54 ± 0.14	0.52 ± 0.14	0.29

volume ratio					

Table S3: Baseline clinical, physiological, and CT characteristics of nasal RNA-seq subgroup

Data are expressed as median (IQR), n (%).

Clinical characteristics	N=237/518
Age (years)	46 (30 to 54)
Age at first diagnosis (years)	16 (6-34)
Duration of disease (years)	22 (13-32)
Sex	
Female	127 (54%)
Male	110 (46%)
BMI (kg/m^2)	26 (23 to 30)
Smoking status	
Current smoker	10 (4%)
Ex-smoker	51 (22%)
Non-Smoker	176 (74%)
GINA	
1	57 (24%)
2	29 (12%)
3	64 (27%)
4	71 (30%)
5	14 (6%)
Inhaled corticosteroids	
No	55 (23%)
Yes	182 (77%)
Systemic corticosteroids	
No	225 (95%)
Yes	12 (5%)
ACQ-6 total mean score	0.67 (0.33-1.33)
SADT score	5.01 (4.34 - 5.70)
MINI AQLQ total mean score	5.9 (5.1-6.4)
EuroQoL 5D-5L	80 (75-90)
Exacerbations over previous 12 months median(IQR)	0 (0-0)
**Inflammatory profile**	
FeNO (single flow at 50ml/s) (ppb)	26 (17-42)
FeNO > 50ppb	
No	189 (80%)
Yes	48 (20%)
Blood Eosinophils (10^9/L)	0.2 (0.1-0.4)
Blood Eosinophils (10^9/L)≥0.3	
No	154 (65%)
Yes	83 (35%)
Blood neutrophils (10^9/L)	3.5 (2.8-4.4)
Sputum Eosinophils (% of non-squamous cells)	0.4 (0.0 - 3.2)
Sputum Eosinophils (% of non-squamous cells)≥3	
No	97 (75%)
Yes	33 (25%)
Sputum Neutrophils (% of non-squamous cells)	51.3 (31.9 - 70.2)
Sputum Macrophages (% of non-squamous cells)	34.6 (19.2 - 57.8)

**Sputum lymphocytes (% of non-squamous cells)**	0.7 (0.3 - 1.8)
**Lung physiology parameters**	
**Spirometry**	
**FEV1 % of predicted normal value**	84.6 (72.4 to 93.9)
**FVC (% predicted of normal value)**	97.7 (88.9 - 106.2)
**FEV1/FVC (%)**	69.4 (62.6 to 76.0)
**FEF2575 (% predicted of normal value)**	56.9 (42.2 to 74.7)
**FEF50 (% predicted of normal value)**	62.9 (47.0 to 81.3)
**Body plethysmography**	
**TLC (L) (% predicted of normal value)**	103.4 (95.9 to 113.5)
**RV/TLC (% predicted of normal value)**	101.4 (88.7 to 119.1)
**Oscillometry**	
**X5 (kPa/L/s)**	-0.14 (-0.20 to -0.10)
**R5 (kPa/L/s)**	0.43 (0.34 - 0.54)
**AX (kPa/L)**	0.54 (0.28 to 1.57)
**R20 (kPa/L/s)**	0.35 (0.30 - 0.40)
**R5-20 (kPa/L/s)**	0.07 (0.02 to 0.15)
**Multiple breath nitrogen washout**	
**Sacin*VT (L^{-1})**	0.11 (0.08 to 0.16)
**Scond*VT (L^{-1})**	0.03 (0.02 to 0.05)
**Quantitative CT**	
**Expiratory MLD (Hounsfield Units (HU))**	-680.1 (-719.7 to -633.1)
**Inspiratory MLD (HU)**	-831.7 (-857.3 to -808.4)
**Expiratory to inspiratory MLD ratio**	0.81 (0.76 - 0.88)
**Expiratory Voxel index (VI) at -856 HU**	6.54 (2.02 - 15.6)
**Inspiratory VI at -950 HU**	3.00 (1.29 - 6.34)
**Median inspiratory % WA/TA**	62.3 (60.9 - 65.1)
**Median inspiratory LA/BSA (mm^2)**	10.3 (8.5 - 12.1)
**Median inspiratory TA/BSA (mm^2)**	27.5 (24.9 - 31.8)
**Median inspiratory WA/BSA (mm^2)**	17.3 (15.4 - 19.9)
**Inspiratory Pi10 (mm)**	7.1 (6.3 - 7.8)
**Inspiratory Po20 (mm)**	7.3 (6.7 to 8.2)
**15th percentile inspiratory (HU)**	-919.0 (-933.4 to -903.4)
**Expiratory lung volume (mL)**	2620.0 (2129.8 - 3021.9)
**Inspiratory lung volume (mL)**	5222.7 (4416.2 - 6394.6)
**Expiratory to inspiratory lung volume ratio**	0.50 (0.43 - 0.57)

Table S4. List of significantly differentially expressed genes in Group 3 (BDR in both) versus Group 1 (spirometry BDR only).

After conducting our differential gene expression in group 3 versus group 1, we extracted significant genes on two main thresholds: log2(FC) > 0.25 or < -0.25; unadjusted p-value <0.05. We present the genes with log2(FC), unadjusted p-values and adjusted p-values with Benjamini-Hochberg correction.

Symbol	Base Mean	Log2FC	p-value	Adjusted p-value
**IDO1**	18.072	-1.347	0.000	0.039
**NDUFV2**	8.154	-0.842	0.000	0.004
**LRRC37A2**	69.112	-0.531	0.000	0.022
**CLEC2D**	17.826	-0.466	0.000	0.039
**NRIP1**	25.398	-0.434	0.000	0.049
**AGFG1**	28.069	-0.416	0.000	0.035
**FAM135A**	132.944	-0.353	0.000	0.035
**PHACTR2**	55.537	-0.343	0.000	0.039
**ARAP2**	64.495	-0.321	0.000	0.049
**DENND1B**	64.672	0.277	0.000	0.049
**RNF19A**	87.668	0.277	0.000	0.049
**SLC15A2**	118.265	0.278	0.000	0.029
**CBLB**	327.208	0.279	0.000	0.039
**PDLIM5**	163.801	0.283	0.000	0.039
**RNF217**	521.868	0.294	0.000	0.044
**MAP2K4**	242.668	0.304	0.000	0.039
**CCPG1**	120.103	0.311	0.000	0.035
**PIN1**	258.696	0.317	0.000	0.025
**PFKM**	193.698	0.321	0.000	0.035
**UBAC1**	119.673	0.348	0.000	0.044
**RAB40B**	164.081	0.362	0.000	0.010
**ZNF358**	88.274	0.365	0.000	0.035
**ZNF517**	76.556	0.374	0.000	0.005
**ANKRD35**	36.112	0.470	0.000	0.044
**LY6G5C**	17.554	0.512	0.000	0.016
**CD300A**	151.503	1.349	0.000	0.041

Table S5. List of significantly upregulated pathways in group 3 versus group 1.

After conducting our differential gene expression analysis, we extracted upregulated genes on two main thresholds: log2(FC) > 0.25; unadjusted p-value < 0.05. These genes were then used for gene enrichment pathway analysis using GO Biological Process 2023, GO Cellular Component 2023 and GO Molecular Function 2023 databases. We present the most significant pathways with p-value and False Discovery Rate

Term	Analysis	p-value	Adj p-value	Odds Ratio	Combined Score	Genes
**Ubiquitin Conjugating Enzyme Binding (GO:0031624)**	MF	0.000	0.008	102.344	847.329	RNF19A; RNF217
**Ubiquitin-Like Protein Conjugating Enzyme Binding (GO:00443900)**	MF	0.000	0.008	83.129	655.676	RNF19A; RNF217
**Ubiquitin Protein Ligase Activity (GO:0061630)**	MF	0.002	0.025	13.689	84.053	RNF19A; RNF217; CBLB
**Ubiquitin-like Protein Ligase Activity (GO:0061659)**	MF	0.002	0.025	13.337	80.929	RNF19A; RNF217; CBLB
**Fructose Binding (GO:0070061)**	MF	0.004	0.028	312.172	1705.224	PFKM
**Ubiquitin-Protein Transferase Activity (GO:0004842)**	MF	0.005	0.028	10.225	54.840	RNF19A; RNF217; CBLB
**Mitogen-Activated Protein Kinase Binding (GO:0031434)**	MF	0.005	0.028	249.725	1318.680	PIN1
**Phosphofructokinase Activity (GO:0008443)**	MF	0.005	0.028	249.725	1318.680	PFKM
**Peptide Transmembrane Transport Activity (GO:1904680)**	MF	0.006	0.029	208.094	1066.850	SCL15A2
**Phosphoserine Residue Binding (GO:00508815)**	MF	0.07	0.030	178.357	890.652	PIN1
**AMP Binding (GO:0016208)**	MF	0.008	0.034	138.708	661.819	PFKM
**MAP Kinase Activity (GO:0004708)**	MF	0.012	0.040	96.010	424.939	MAP2K4
**Muscle Alpha-Actinin Binding (GO:0051371)**	MF	0.012	0.040	96.010	424.939	MAP2K4
**Cadherin Binding Involved in Cell-Cell Adhesion (GO:0098641)**	MF	0.014	0.045	77.996	330.974	PDLIM5
**Actinin Binding (GO:0042805)**	MF	0.017	0.046	65.671	268.079	PDLIM5
**Solute:Proton Symporter Activity (GO:0098641)**	MF	0.017	0.046	65.671	268.079	SCL15A2
**Alpha-Actinin Binding (GO:0051393)**	MF	0.019	0.050	56.707	223.630	PDLIM5
**Positive Regulation of Protein Dephosphorylation (GO:0035307)**	BP	0.000	0.064	85.815	682.020	CD300A; PIN1

Table S6. **List of significantly downregulated pathways in group 3 versus group 1**.

After conducting our differential gene expression analysis, we extracted downregulated genes on two main thresholds: log2(FC) > 0.25; unadjusted p-value < 0.05, and entered them into gene enrichment pathway analysis using GO Biological Process 2023, GO Cellular Component 2023 and GO Molecular Function 2023 databases.

Term	Analysis	p-value	Adjusted p-value	Odds Ratio	Combined Score	Genes
**Positive Regulation of Lymphocyte Apoptotic Process (GO:0070230)**	BP	0.003	0.035	499.650	2955.690	IDO1
**Tryptophan Catabolic Process (GO:0006569)**	BP	0.003	0.025	416.354	2398.853	IDO1
**Tryptophan Metabolic Process (GO:0006568)**	BP	0.003	0.025	416.354	2398.853	IDO1
**Kynurenine Metabolic Process (GO:0070189)**	BP	0.004	0.025	356.857	2008.476	IDO1
**Positive Regulation of T Cell Apoptotic Process (GO:0070234)**	BP	0.004	0.025	312.234	1720.615	IDO1
**Aromatic Amino Acid Family Catabolic Process (GO:0009074)**	BP	0.004	0.025	277.528	1500.174	IDO1
**Regulation of T Cell Apoptotic Process (GO:0070232)**	BP	0.005	0.025	249.762	1326.334	IDO1
**Striated Muscle Tissue Development (GO:0014706)**	BP	0.006	0.027	192.096	973.893	NDUFV2
**NADH Dehydrogenase Activity (GO:0003954)**	CC	0.003	0.030	416.354	2398.853	NDUFV2
**Nuclear Glucocorticoid Receptor Binding (GO:0035259(**	CC	0.004	0.030	356.857	2008.476	NRIP1
**Nuclear Retinoid X Receptor Binding (GO:0046965)**	CC	0.004	0.030	277.528	1500.174	NRIP1
**Cellular Response to Estradiol Stimulus (GO:0071392)**	BP	0.009	0.036	131.395	619.440	NRIP1
**Nuclear Retinoic Acid Receptor Binding (GO:0042974)**	CC	0.008	0.036	156.055	760.963	NRIP1
**Oxidoreductase Activity, Single Donors, Incorporation of Two Oxygen Atoms (GO:0016702)**	CC	0.009	0.036	124.819	582.373	IDO1
**NADH Dehydrogenase (Ubiquinone) Activity (GO:0008137)**	CC	0.015	0.036	77.965	328.712	NDUFV2
**NADH Dehydrogenase (Quinone) Activity (GO:0050136)**	CC	0.015	0.036	75.598	316.494	NDUFV2
**Nuclear Estrogen Receptor Binding (GO:0030331)**	CC	0.015	0.036	75.598	316.494	NRIP1
**Phosphatidylinositol-3,4,5-Triphosphate Binding (GO:0005547)**	CC	0.016	0.036	71.271	294.333	ARAP2
**Alpha-Amino Acid Catabolic Process (GO:1901606)**	BP	0.013	0.041	89.121	387.191	IDO1
**Response to Estradiol (GO:0032355)**	BP	0.014	0.041	83.171	355.828	NRIP1
**Regulation of Activated T Cell Proliferation (GO:0046006)**	BP	0.015	0.041	77.965	328.712	IDO1
**Mitochondrial Electron Transport, NADH to Ubiquinone (GO:0006120)**	BP	0.015	0.041	75.598	316.494	NDUFV2
**Amino Acid Catabolic Process (GO:0009063)**	BP	0.017	0.042	67.412	274.777	IDO1

**Oxidoreduction-Driven Active Transmembrane Transport Activity (GO:0015453**	CC	0.024	0.048	47.024	175.298	NDUFV2

References

[1] Schiphof-Godart L, van der Wiel E, ten Hacken NH, et al. Development of a tool to recognize small airways dysfunction in asthma (SADT). Health Qual Life Outcomes 2014;12:155. https://doi.org/10.1186/s12955-014-0155-7.

[2] Miller MR, Hankinson J, Brusasco V, et al. Standardisation of spirometry. Eur Respir J 2005;26:319–38. https://doi.org/10.1183/09031936.05.00034805.

[3] Quanjer PH, Stanojevic S, Cole TJ, et al. Multi-ethnic reference values for spirometry for the 3-95-yr age range: the global lung function 2012 equations. Eur Respir J 2012;40:1324–43. https://doi.org/10.1183/09031936.00080312.

[4] GINA. Global Initiative for Asthma. Global Strategy for Asthma Management and Prevention 2014. 2014.

[5] Thamrin C, Robinson PD, Farah CS, et al. Technical standards for respiratory oscillometry and bronchodilator response cut-offs. Eur Respir J 2022;59:2102663. https://doi.org/10.1183/13993003.02663-2021.

[6] Oostveen E, Boda K, van der Grinten CPM, et al. Respiratory impedance in healthy subjects: baseline values and bronchodilator response. Eur Respir J 2013;42:1513–23. https://doi.org/10.1183/09031936.00126212.

[7] Johansson H, Wollmer P, Sundström J, et al. Bronchodilator response in FOT parameters in middle-aged adults from SCAPIS: normal values and relationship to asthma and wheezing. Eur Respir J 2021;58:2100229. https://doi.org/10.1183/13993003.00229-2021.

[8] Jetmalani K, Brown NJ, Boustany C, et al. Normal limits for oscillometric bronchodilator responses and relationships with clinical factors. ERJ Open Res 2021;7:00439–2021. https://doi.org/10.1183/23120541.00439-2021.

[9] Sterk PJ, Fabbri LM, Quanjer PH, et al. Airway responsiveness. Standardized challenge testing with pharmacological, physical and sensitizing stimuli in adults. Report Working Party Standardization of Lung Function Tests, European Community for Steel and Coal. Official Statement of the European Respiratory Society. Eur Respir J Suppl 1993;16:53–83.

[10] Wanger J, Clausen JL, Coates A, et al. Standardisation of the measurement of lung volumes. Eur Respir J 2005;26:511–22. https://doi.org/10.1183/09031936.05.00035005.

[11] Postma DS, Brightling C, Baldi S, et al. Exploring the relevance and extent of small airways dysfunction in asthma (ATLANTIS): baseline data from a prospective cohort study. Lancet Respir Med 2019;7:402–16. https://doi.org/10.1016/S2213-2600(19)30049-9.

[12] Dobin A, Davis CA, Schlesinger F, et al. STAR: ultrafast universal RNA-seq aligner. Bioinformatics 2013;29:15–21. https://doi.org/10.1093/bioinformatics/bts635.
